# Hepatotoxicity of Herbal Supplements Mediated by Modulation of Cytochrome P450

**DOI:** 10.3390/ijms18112353

**Published:** 2017-11-08

**Authors:** Christopher Trent Brewer, Taosheng Chen

**Affiliations:** 1 Department of Chemical Biology and Therapeutics, St. Jude Children’s Research Hospital, Memphis, TN 38105, USA; Christopher.Brewer@STJUDE.ORG; 2 Integrated Biomedical Sciences Program, University of Tennessee Health Science Center, Memphis, TN 38163, USA

**Keywords:** cytochrome P450, pregnane X receptor, constitutive androstane receptor, herbal supplement, drug-induced liver injury, herb-induced liver injury, drug-drug interactions, drug-herb interactions, herb-herb interactions, xenobiotic metabolism

## Abstract

Herbal supplements are a significant source of drug-drug interactions (DDIs), herb-drug interactions, and hepatotoxicity. Cytochrome P450 (CYP450) enzymes metabolize a large number of FDA-approved pharmaceuticals and herbal supplements. This metabolism of pharmaceuticals and supplements can be augmented by concomitant use of either pharmaceuticals or supplements. The xenobiotic receptors constitutive androstane receptor (CAR) and the pregnane X receptor (PXR) can respond to xenobiotics by increasing the expression of a large number of genes that are involved in the metabolism of xenobiotics, including CYP450s. Conversely, but not exclusively, many xenobiotics can inhibit the activity of CYP450s. Induction of the expression or inhibition of the activity of CYP450s can result in DDIs and toxicity. Currently, the United States (US) Food and Drug Administration does not require the investigation of the interactions of herbal supplements and CYP450s. This review provides a summary of herbal supplements that inhibit CYP450s, induce the expression of CYP450s, and/or whose toxicity is mediated by CYP450s.

## 1. Introduction

**Figure 1:**
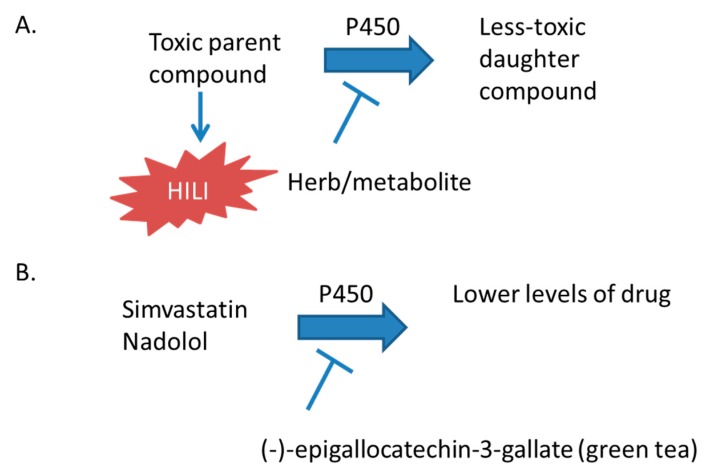
The effect of P450 inhibitors on drug toxicity. The parent compound can be the herbal supplement itself or a co-administered Food and Drug Administration (FDA)-approved drug. (**A**) Schematic of the general mechanism; (**B**) a specific example discussed in the text [5].

The aim of this review is to highlight the effects of herbal supplement use on xenobiotic metabolism and the development of hepatotoxicity or modulation of therapeutic efficacy, either alone or with concurrent use of other herbal supplements and Food and Drug Administration (FDA)-approved pharmaceuticals. Cytochrome P450 (CYP450) enzymes metabolize a large number of xenobiotics, which may lead to the formation of hepatotoxic compounds. Fortunately, pharmaceuticals are evaluated for the potential to inhibit or induce CYP450 enzymes before being marketed in the United States (US) [1]. However, regulations for herbal supplements in the United States do not require surveillance or the reporting of adverse events by the manufacturer to the FDA [2]. Thus, the data concerning the hepatotoxicity of herbal supplements are derived from case reports and series, retrospective databases, and progressive registries, such as the US Drug-Induced Liver Injury (DILI) Network and the Spanish DILI Registry [3,4]. Therefore, herbal supplements that inhibit CYP450 enzymes are problematic, because in addition to affecting the therapeutic efficacy of drugs that require bioactivation for effect, these interactions may inhibit the metabolism of toxic parent compounds to less toxic daughter compounds (Figure 1) [5].

**Figure 2:**
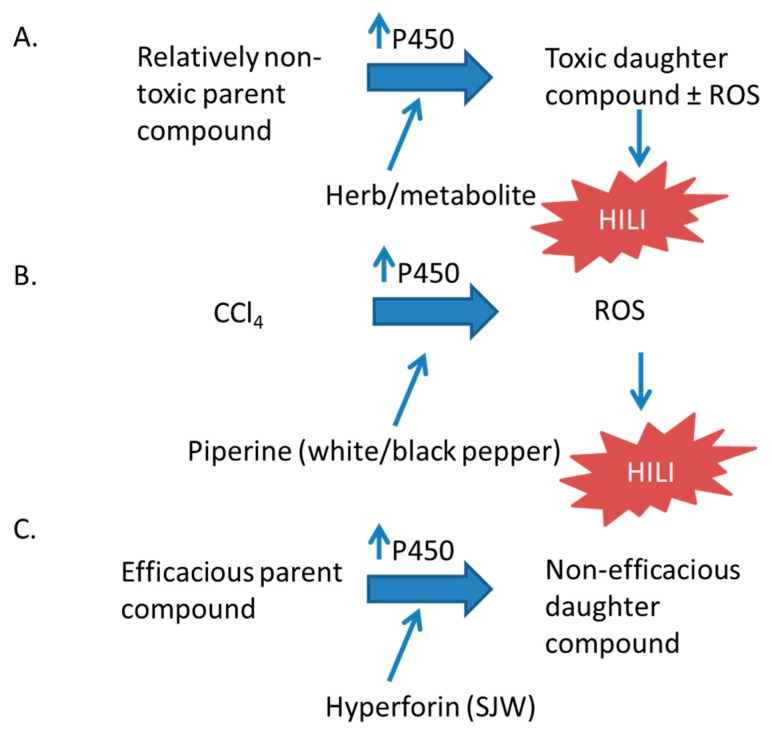
The effect of P450 inducers on toxicity. The parent compound can be the herbal supplement itself or a co-administered FDA-approved drug. (**A**) Schematic of the general mechanism; (**B**,**C**) specific examples discussed in the text [10,11,12,13,14].

The expression of CYP450 enzymes is induced by nuclear receptors that respond to xenobiotics. Many CYP450 enzymes are transcriptionally regulated by the xenobiotic receptors pregnane X receptor (PXR, also known as NR1I2, SXR, or PAR) and constitutive androstane receptor (CAR; NR1I3) [6,7,8,9]. We will discuss the involvement of PXR and CAR in herbal supplement hepatotoxicity later in this review. Many herbal supplements can activate these receptors leading to an increased expression of CYP450 enzymes and can affect the metabolism of other xenobiotics. In many instances of this can lead to hepatotoxicity, as well as deactivating active drugs and decreasing the therapeutic effect. The induction of CYP450 enzymes may result in hepatotoxicty by increasing the metabolism of less toxic parent compounds to much more toxic daughter compounds (Figure 2) [10,11,12,13,14].

**Figure 3:**
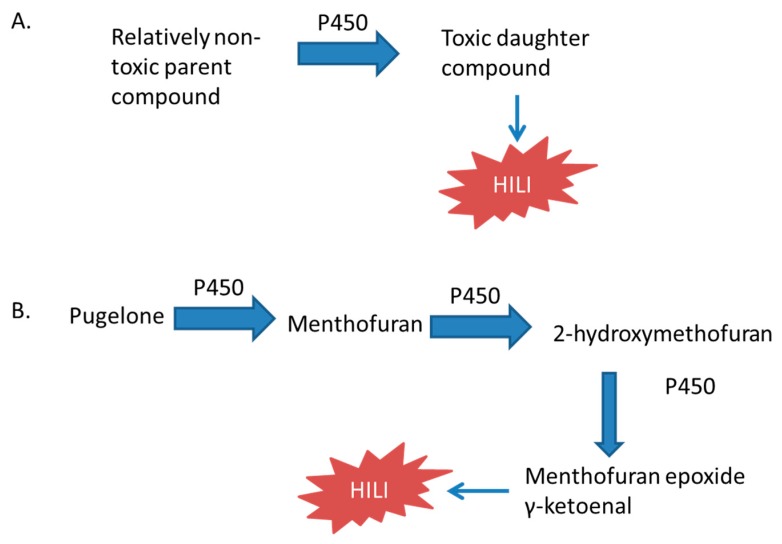
The effect of compounds metabolized by the P450 system on toxicity. The parent compound can be the herbal supplement itself or a co-administered FDA-approved drug. (**A**) Schematic of the general mechanism; (**B**) a specific example discussed in the text [22,23,24].

Concurrent use of pharmaceuticals with herbal supplements, as well as the use of multiple herbal supplements, presents additional problems. Many patients do not report herbal supplement use to their physicians [15,16,17,18], with one study showing that approximately one-third of patients do not disclose the use of herbal supplements to their physicians [19]. Complicating matters further, a Spanish study shows that 20% of patients use herbal supplements concurrently with prescription drugs [20], with another study showing that 20% of patients reported the use of one or more herbal supplements, and 30% of patients reported taking an herbal supplement to treat the same condition that they were concomitantly taking prescription medication to treat [21]. Combining herbal supplements with prescription drugs without the knowledge of primary care providers is especially troubling when considering that the hepatotoxicity of herbal supplements can also arise from herb-drug interactions (Figure 3) [22,23,24].

## 2. Literature Search Methodology

To develop this review concerning the association of herbal supplement use with P450 modulation and hepatotoxicity, we used the search terms “herbal supplement” or “herb” combined with “hepatotoxicity” or “liver injury” to search the PubMed database. From this initial search, we selected compounds associated with hepatotoxicity in an in vivo model. We then searched for each of these supplements with “P450”, “CYP450”, or “cytochrome P450”. Finally, we searched for “herbal supplement” or “herb” combined with “P450”, “CYP450”, or “cytochrome P450”. Care is taken to include reports where the effect (P450 modulation or hepatotoxicity) is attributable to a single causative compound or when an extract of defined herbal ingredients is provided.

## 3. Herbal Supplements with Potential P450-Associated Hepatotoxicity

The most commonly used herbal supplements in the United States are echinacea, garlic, ginko biloba, saw palmetto, ginseng, grape seed extract, green tea, and St. John’s wort [25]. This review will discuss herbal supplements that inhibit cytochrome P450 enzymes (Figure 1) or induce P450 expression (Figure 2), and those whose toxicity is mediated by the P450 system (Figure 3). Examples of these herbal supplements are discussed in the proceeding sections and are summarized in Table 1, Table 2 and Table 3, respective to figure numbering. These interactions can lead to HILI with the use of herbal supplements alone or when used in combination with traditional FDA-approved pharmaceuticals.

### 3.1. Herbal Supplement Inhibition of P450s

Cytochrome P450 enzymes, predominantly hydroxylate xenobiotics and endobiotics, enable conjugation to additional chemical moieties to facilitate elimination from the body [26]. In the case of hepatotoxic compounds, these enzymes decrease the exposure of the compound to the liver (Figure 1). Therefore, when patients are administered multiple xenobiotics, care should be taken that one compound does not increase the toxicity of another by inhibiting the metabolism of a toxic compound to its less-toxic daughter compounds. The ability of pharmaceuticals to inhibit P450 enzymes is evaluated during drug development and is usually well characterized before reaching market and general patient populations [1]. However, in the United States, herbal supplements are not required to be evaluated for this effect before reaching market. This would be problematic even if only the activation of inactive pro-drugs was prevented and the therapeutic efficacy was decreased. Inhibition of CYP450 enzymes may prevent the activation required for efficacious treatment by inactive prodrugs (e.g., tamoxifen) [27]. However, this is very concerning because of the potential for increasing hepatic and systemic exposure to toxic compounds, which would otherwise be metabolized by the P450 system. For example, catechins in green tea, terpenes in black cohosh and cranberry, geniposide and genipin in *Gardenia*, fucomarins in grapefruit juice, and *Echinacea* extract may inhibit the activity or decrease the expression of cytochrome P450 enyzmes (Table 1).

#### 3.1.1. Green Tea

Green tea is traditionally made in China from the leaves of *Camellia sinensis,* and it is consumed to treat cancer, cardiovascular disease, dyslipidemia, inflammation, and weight loss [56,57,58,59,60]. Green tea use has been associated with hepatotoxicity at higher doses [61,62]. The hepatotoxicity of green tea in humans has been described as exhibiting a hepatocellular pattern of toxicity, and was evaluated by using the Roussel Uclaf Causality Assessment Method (RUCAM) causality assessment scale [62]. Additionally, using green tea in combination with other supplements was associated with liver injury that was shorter-onset and more-serious than that observed when patients were taking green tea alone [62]. This more-serious toxicity could be the result of interactions between the green tea and other components of the preparations. Whole green tea extract and the catechin (−)-epigallocatechin-3-gallate administered in a purified form inhibit the activity of multiple cytochrome P450 enzymes, including CYP2B6, CYP2C8, CYP2C19, CYP2D6, and CYP3A, in human liver and intestinal microsomes [30]. In rats that were administered commercially available green tea, the activities of hepatic microsomal cytochrome P450s were decreased, including those of CYP2C, CYP2E1, and CYP3A [63]. (−)-Epigallocatechin-3-gallate administered at non-lethal doses to mice decreased the levels of superoxide dismutase, catalase, and glutathione peroxidase. In mice, lethal hepatotoxicity was observed at higher doses [64]. Toxicity attributed to green tea extract has also been reported in rats [65]. Green tea extract administered to rats affected the pharmacokinetics of simvastatin and inhibited the hydroxylation of midazolam by CYP3a in liver microsomes [31]. Green tea extract and (−)-epigallocatechin-3-gallate pre-treatment reduced the area under the time-plasma concentration curve (AUC) and maximum plasma concentration (Cmax) of nadolol—a β-blocker that is not metabolized by cytochrome P450 enzymes but is reported to be a substrate for several drug transporters—in rats [5]. Thus, the consumption of green tea may inhibit the intestinal absorption of pharmaceutics taken concurrently. Additionally, green tea extract and a component of this extract, (−)-epigallocatechin-3-gallate, inhibit multiple P450 enzymes, and this inhibition may contribute to the toxicity that is associated with green tea use. Please see an earlier review concerning the hepatotoxicity of green tea for further information [66].

#### 3.1.2. Black Cohosh

Black cohosh (*Actae*/*Cimicfuga racemose*) is native to the eastern region of North America. The roots and rhizomes of black cohosh are used as a hormone replacement and as an anti-inflammatory [67,68]. Hepatotoxicity has been reported in patients taking black cohosh [69,70]. Black cohosh has been reported as a probable causation of liver injury [69,70,71]. Although, a prior review discounted previous separate reports of hepatotoxicity that is attributed to black cohosh due to the difficulty in assigning causation as a result of using mixtures of multiple supplements, the temporal association of consumption and toxicity, and the presence of other confounding variables [72]. However, a recent case report found black cohosh to be a highly probable cause of hepatotoxicity [71]. In humans, the presence of 4HNE protein adducts in the hepatocytes of patients administered black cohosh was associated with acute liver necrosis and pathologic changes indicative of autoimmune hepatitis [70]. In mice, black cohosh administration increased liver weight and both the expression and activity of Cyp2b and Cyp3a [73]. However, this induction seems to be mediated by mouse but not by human PXR. Compounds isolated from the extract of black cohosh were triterpene glycosides, fukinolic acid, cimicifugic acid A, and cimicifugic acid B, which all inhibited multiple CYP450 enzymes (1A2, 2D6, 2C9, 3A4) [29]. Although these data suggest the potential for interaction with drugs that are metabolized by these enzymes, the authors found that these compounds were not directly toxic to the human hepatoma cell line HepG2, supporting the hypothesis that black cohosh causes an immune-mediated liver injury [29]. Black cohosh is hepatotoxic to humans, and components of black cohosh inhibit P450 enzymes; but, whether this toxicity is modulated by interactions with the P450-inhibitory compounds in the extract is unclear.

#### 3.1.3. Cranberry

Cranberry (*Vaccinium macrocarpon*) is native to North America and is consumed to treat wounds, prevent urinary tract infections, and to treat diabetes [74,75,76]. The triterpenes, marslinic acid, corosolic acid, and ursolic acid that are present in cranberry juice inhibit CYP3A4 in human intestinal microsomes [43]. Cranberry extract also inhibited CYP1A2, CYP2D6, and CYP3A4 [44].

#### 3.1.4. Grapefruit

The grapefruit (*Citrus paradisi*) is the result of an accidental cross between the pomelo (*C*. *grandis*) and the sweet orange (*C*. *sinesis*) that is believed to have originated in Barbados [77]. Capsules containing grapefruit extract are used to treat hypercholesterolemia, weight reduction, asthma, atherosclerosis, cancer, and depression [78,79,80,81,82]. Furocoumarins, dihydroxy bergamottin, and gergamottin found in grapefruit juice inhibit CYP3A4 activity in human liver microsomes [38,39]. Grapefruit juice also inhibits organic anion-transporting polypeptide [39]. In humans, the activity of clopidogrel, an antiplatelet drug used for coronary artery disease that requires activation by CYP2C19 and CYP3A4, is decreased by grapefruit juice [83].

#### 3.1.5. *Echinacea*

*Echinacea* is a genus of flowering plants found in the Eastern and Midwestern US. It is used to treat colds, upper respiratory infections, and dermatologic issues, and contains cichoric acid, caftaric acid, and echinacoside [84,85,86]. Crude extracts of *Echinacea* activate hPXR to induce CYP3A4 gene transcription and increase the mRNA of CYP1A2, CYP3A4, and MDR1 in the HepG2 cell line [87]. However, Echinacea extract may inhibit the rifampicin-mediated induction of CYP3A4 transcription via hPXR [45] and has been reported to inhibit CYP1A2 and CYP3A [41,42].

#### 3.1.6. *Gardenia*

Plants of the *Gardenia* genus are native to Africa, southern Asia, Australia, and Oceania. Geniposide is an iridoid glycoside found in the *Gardenia jasminoides* fruit. The fruit is used as a food coloring, an anti-inflammatory, an antithrombotic, and as an antidepressive [88,89,90]. Rats treated with geniposide or the crude extract of *G*. *jasminoides* had a decreased hepatic CYP3A4 expression and activity that was associated with an increase in glutathione [34]. Genipin, which is also found in *G*. *jasminoides* fruit, induces CYP2D6 and inhibits CYP2C19 and CYP3A4 mRNA, protein, and activity in the human hepatoma cell line HepG2. Genipin also decreased the expression of MDR1 (P-gp) [35]. Geniposide decreased hepatic CYP3a levels in rat livers [34]. The administration of gardenia yellow color caused hepatoxicity in rats [91]. Administering Gardenia yellow color, consisting of 30% geniposide (*w*/*v*), caused increased aspartate transaminase (AST) and alanine aminotransferase (ALT) in rats and histologic changes in rat liver [91]. The aqueous and alcoholic extract of *Fructus gardeniae* and geniposide all cause hepatoxicity in rats [92,93]. Cranberry, Grapefruit, *Echinacea*, and *Gardenia* alone may not be associated with toxicity in humans, but their effects upon the P450 system warrant concern.

### 3.2. Herbal Supplement Induction of P450 Enzymes

The hydroxylation of xenobiotics and endobiotics by P450 enzymes can produce daughter compounds that are more toxic than the parent compound (Figure 2). This is problematic enough when the efficacy of pharmaceuticals is affected by decreasing plasma concentrations of drugs, especially in highly active anti-retroviral therapy regimens in which one component of the drug may increase the metabolism of another component and decrease the efficacy of anti-HIV treatment [94,95]. In the case of P450-mediated formation of toxic metabolites, this effect may be fatal. Fortunately, pharmaceuticals are evaluated for this potential. However, in the United States, herbal supplements are not required to be evaluated for their potential to induce the P450 system [1]. Hyperforin in St. John’s wort, multiple compounds in *Gingko biloba*, piperine in black and white pepper, diallyl sulfide in garlic, and grapeseed extract increase p450 expression (Table 2).

#### 3.2.1. The Pregnane X Receptor (PXR)

Activation of PXR by ligands such as rifampicin and many other compounds results in the transcription of genes that are involved in the transport and metabolism of xenobiotics. PXR is a nuclear hormone receptor (NHR), a class of proteins that are characterized by a DNA-binding domain, as well as a ligand-binding domain (LBD). The LBD of human PXR (hPXR) binds to many ligands with a wide range of different structures [108,109]. NHRs bind target DNA sequences in the promoters of target genes to induce their transcription. The LBD of PXR interacts with agonists to enable the recruitment of co-activating proteins to trigger the transcriptional activation [108,109,110,111]. PXR regulates the expression of cytochrome P450 enzymes (CYPs) CYP3A4, CYP2B6, CYP2C9, and CYP2C19; phase II enzymes, including UDP-glucuronosyltransferases and sulfotransferases; and transporters, including ATP–binding cassette transporter ABCB1 (also known as MDR1 or P-gp), multiple organic anion transporters, and multidrug-resistance protein 3 (MRP3) [6,7,112,113,114]. The hPXR protein is expressed in the liver and intestines [115]. A mouse model in which mouse PXR is replaced with hPXR enables examination of the effect of hPXR-specific ligands on hPXR function in vivo [116,117]*.*

#### 3.2.2. The Constitutive Androstane Receptor (CAR)

CAR also controls the expression of xenobiotic metabolizing enzymes and transporters. Unlike PXR, CAR is constitutively active in the absence of ligand [118]. Agonist binding to CAR further activates CAR and results in the activation of such target genes as CYP2B6, the CYP2C subfamily, and CYP3A4 [9]. CAR also controls the expression of other genes that are involved in drug metabolism and transport [119,120]. Phenobarbital (used for refractory seizure conditions) induces CAR’s dephosphorylation, which indirectly activates CAR and increases the transcription of target genes [9,121]. Although 1,4-bis[2-(3,5-dichloropyridyloxy)]benzene activates mouse CAR by direct binding, it does not affect human CAR [122]. Conversely, 6-(4-chlorophenyl)imidazo(2,1-b)(1,3)thiazole-5-carbaldehyde *O*-(3,4-dichlorobenzyl)oxime binds to and activates human CAR, but not mouse CAR [118,123]. Therefore, to study the in vivo effects of human CAR ligands, a humanized transgenic mouse model is used.

#### 3.2.3. St. John’s Wort

St. John’s wort (*Hypericum perforatum*) is used to treat anxiety and depression. St. John’s wort is native to Europe, but is now also cultivated in the US, Canada, and Australia [124]. A constituent of St. John’s wort, hyperforin, increases the expression of CYP2C9 [99] and CYP3A4 [100] via activation of the hPXR. The concentration in commercial preparations of hyperforin can vary up to 10-fold between sources [100]. The use of St. John’s wort can increase the metabolism of compounds and result in the formation of toxic products. Indeed, there has been a report of *Hypericum perforatum* induced liver injury that is associated with the use of copaiba, a herbal supplement that is used as an anti-inflammatory [125]. Whether this is due to hPXR activation is unclear. Although St. John’s wort is not reported to be toxic alone, it may be responsible for increasing the toxicity of compounds that an organism is exposed to simultaneously. This toxicity may be mediated by the induction of the P450 system by St. John’s wort constituents, such as hyperforin, activating hPXR.

#### 3.2.4. Gingko Biloba

*Gingko biloba* is native to China but is now cultivated in many regions. It is used as an anti-hypertensive as well as to treat macular degeneration and tinnitus [126,127,128,129]. *Gingko biloba* extract increases hepatocyte DNA replication (an early indicator of hypertrophic change) and Cyp2b10, Cyp1a1, and Cyp3a11 mRNA in WT mice, but not in Car^−/−^ mice [130]. This discrepancy suggests that the induction of the P450 system by mCar is associated with hepatic hypertrophy that is secondary to *Ginkgo biloba* consumption. *Ginkgo biloba* extract activates the transcription of a CYP3A4 promoter when an expression construct is overexpressed with either mouse or human PXR in HepG2 cells [101]. *Ginkgo biloba* extract also increases the expression of CYP3A4, CYP3A5, and ABCB1 (MDR1) mRNA in LS180 colorectal cancer cells [101]. Further characterization of specific components of *Gingko biloba* extract demonstrated that ginkgolide A and ginkgolide B induce the expression of CYP2B6, CYP3A4, UGT1A1, MDR1, and MRP2 mRNA in primary human hepatocytes [102]. Ectopically overexpressed hPXR is activated by both ginkgolide A and B in HepG2 [102]. *Ginkgo biloba* is associated with hepatotoxicity in humans [61]. Whole extract of *Ginkgo biloba,* as well as the isolated compounds gingkolide A and B themselves activate hPXR, thereby increasing the expression of CYP3A4, CYP3A5, and CYP2B6 and other genes controlled by hPXR. Whether the toxicity reported by humans taking *Ginkgo biloba* is a result of hPXR activation or of induction of the P450 system is unknown.

#### 3.2.5. Ginseng

Ginseng is found in North America (*Panax quinquefolius*) and in eastern Asia (*Panax ginseng*). The roots and leaves are available as pills and in energy drinks, teas, and coffee beverages. *Panax quinquefolius* is used to reduce the incidence and severity of colds, whereas *Panax ginseng* is believed to enhance cognitive ability and to lower blood sugar levels; both contain ginsenosides and gintonin [97,131,132]. Wild *Panax ginseng* extract inhibits the induction of Cyp1a1 mRNA and protein expression induced by benzo-pyrene. Cyp1a1 metabolizes benzypyrene, and this activation is required for toxicity [133]. This activation is protective against benzo-pyrene–mediated toxicity in rats [133]. However, in clinical situations in which P450 activation results in the decrease of a toxic parent compound and the formation of a relatively non-toxic daughter compound, ginseng may contribute to toxicity (Figure 1). Such may have been the case in a patient administered imatinib [134]. The patient took imatinib daily for seven years with no complications, but after self-administering *Panax ginseng* as a constituent of an energy drink for three months, there may have been an interaction between imatinib and the ginseng that resulted in liver injury [135]. CYP3A4 and CYP3A5 metabolize 20(*S*)-protopanaxadiol, a ginseng sapogenin, in human liver and intestinal microsomes [97,136]. Additionally, several ginsenosides affect hPXR: Ginsenoside F2 and protopanaxadiol activate hPXR to induce the transcription of the CYP3A4 gene, whereas panaxotriol, Rg2, pseudoginsenoside F11, Rg1, ginsenoside, and Rb3 inhibt hPXR [97]. The potential for interactions and toxicity with ginseng administration may result from the ability of ginseng’s components to affect the expression of P450 enzymes.

#### 3.2.6. Piperine

Piperine is an alkaloid that is responsible for the flavor and smell of black and white pepper (*Piper nigrum*). White and black pepper are taken for GI distress, bronchitis, malaria, cholera, and cancer [137,138]. The piperamides in *P*. *nigrum* are thought to cause ROS production and to cause oxidative damage in cancer cells [139]. Piperine binds to and activates hPXR to induce the expression of CYP3A4 and MDR1 [98]. When administered simultaneously with CCl_4_, piperine increased the amounts of plasma liver enzymes, hepatic lipid peroxidation, and NADPH-cytochrome c reductase activity [10]. The toxicity of CCl_4_ may be due to P450-mediated ROS production [140]. The increased activity of the P450 system due to hPXR activation by piperine may potentiate the toxicity associated with CCl_4_ administration. Further studies of hPXR transgenic mice may fully elucidate the role of piperine in CCl_4_-mediated HILI.

#### 3.2.7. Garlic

Garlic (*Allium sativum*) is used to treat a variety of medical conditions. Consumption of large amounts of garlic is believed to reduce the number of bites by insects, such as ticks [141]. Several linear sulfur-containing compounds are found in garlic oil, including diallyl sulfide, diallyl disulfide, and diallyl trisulfide [142]. Intraperitoneal injection of diallyl sulfide increased the expression of Cyp2b1 and Cyp2b2 mRNA in rat livers [107]. Diallyl sulfide induced Cyp2b10 mRNA in WT mice, and, to a lesser extent, in Car^−/−^ mice [142]. Dially sulfide also increased Sult1e1 mRNA and protein expression as well as Car nuclear localization in the livers of WT mice but not in those of Car^−/−^ mice [143]. Orally administered fresh garlic homogenate causes hepatotoxicity in rats [144,145]. Further studies using Car mouse models would reveal whether Car activation by garlic homogenate or diallyl sulfide is required for the observed toxicity.

### 3.3. Herbal Supplement Hepatotoxicity Mediated by the P450 System

In addition to affecting the toxicity of pharmaceuticals, the P450 system can mediate the toxicity of herbal supplements (Figure 3). Most of the compounds discussed here are terpenes, pyrrolizidine alkaloids, and ginsenosides (Table 3). These compounds are found in several herbal supplements that, in many instances, are combined with herbal supplements and pharmaceuticals that modulate the activity and expression of P450 enzymes. Pyrrolizidine alkaloids (PAs) are found in many different species that are consumed as herbal supplements [146]. Ginsenosides are found in ginseng [147]. Examples of terpenes include peppermint oil, menthol, camphor, germander, and pennyroyal.

#### 3.3.1. Characterization of Liver Injury by Pathophysiology

The clinicopathological characterization of DILI (reviewed in Wang et al., 2014) represents the clinical presentation of toxicity and the pathological effects of the injury and is a necessary step to assigning causation to a particular agent [172]. Cholestatic liver injury may be caused by the disruption of intracellular actin or transporter proteins ultimately resulting in bile stasis [173,174]. Hepatocellular liver damage is associated with compounds that are directly toxic to liver parenchyma and ALT elevation. ALT is expressed throughout the liver and is associated with diffuse hepatocellular liver injury [175]. Hepatotoxicity from herbal supplements is predominantly hepatocellular; 12% of patients experience severe, fatal DILI, 4% require liver transplant, and chronic injury develops in 10% [176,177]. Cholestatic liver injury is defined by elevated levels of serum alkaline phosphatase (ALP), γ-glutamyl transferase (GGT), and bilirubin [175]. This subtype is defined by an impeded bile flow and the deposition of bile acids within the liver lobules. Direct damage of biliary epithelium or impaired bile efflux transport can produce cholestasis [178]. Cholestasis can lead to fibrotic change and even hepatic necrosis. ALP is expressed in biliary epithelial cells and is released during biliary obstruction [179]. Mixed liver injury results in cases in which combinations of agents are used or when a single agent has a toxicity mechanism that damages multiple hepatic cell types [180].

#### 3.3.2. Characterization of Liver Injury by Pathogenesis

Characterization of liver injury by pathogenesis considers intrinsic versus idiosyncratic types of injury [181]. Intrinsic liver injury is dose-dependent and in many cases is caused by the parent compound or a metabolite of the parent compound. Intrinsic liver injury associated with a measurable species in the blood or liver [182,183]. These reactions are predictable (i.e., occurring in animal models) and are usually detected in the preclinical development of pharmaceuticals. The pathogenesis of intrinsic liver injury is mediated by the compound and is usually reproducible in laboratory animals [181,183,184,185,186]. Therefore, the pathogenesis of intrinsic liver injury is more easily studied than idiosyncratic liver injury. In the more common form of liver injury, idiosyncratic liver injury, the modification of compounds by the P450 system can result in the formation of protein adducts that are recognized as antigens [187,188,189]. In many cases, these adducts are not detectable in the blood and this effect is not reproducible in laboratory animals [190]. Idiosyncratic liver injury likely results from a combination of genetic and environmental factors that are not reproducible in laboratory animals [181,191]. Unlike intrinsic liver injury, the pathogenesis of idiosyncratic liver injury is not unique to the compound initiating the reaction, but is dependent upon an immune response [192,193]. This type of injury can only be detected in clinical development, but is more commonly only detected after the compound is marketed, due to a relatively low incidence hiding the effect in small study populations.

#### 3.3.3. Assessment of Causality

Predicting the risk of liver injury due to herbal supplements requires both a standardized causality assessment method and a repository of this information reported in the clinic. The Council for International Organizations of Medical Sciences (CIOMS), Working Group IX, developed the Roussel Uclaf Causality Assessment Method (RUCAM), a list of tools and guidelines for researchers and clinicians to evaluate and manage the risk of medicinal products (including herbal supplements) [25,194,195]. These standardized tools were developed to define and assess causality of pharmaceutics in causing DILI. The guidelines were developed to standardize the evaluation of DILI during development and after the public release of pharmaceuticals. The application of this method requires classifying liver injury according to pathophysiology, time to onset, liver tests, historical risk factors, concomitant drug and herb use, patient history, and the response to re-exposure of the compound. The original RUCAM addressed the lack of defined items that had previously resulted in a lack of consensus between evaluators and also introduced the consideration of chronological data. This original version was plagued by intra- and inter-observer variability, as well as a lack of consideration of differential diagnoses due to non-drug/herb causes [195,196,197]. An update to RUCAM addressed these weaknesses and streamlined the evaluation of these cases. The updated definitions of the classification items reduces the variability of assessments and the dependence upon outside experts not involved with the case [195].

#### 3.3.4. Peppermint Oil, Pennyroyal, and Menthol

Menthol is prepared from corn mint or peppermint, or is made synthetically. Menthol is administered topically to treat minor muscle and joint pain by causing the skin to feel cool and then warm, distracting patients from feeling deeper pain [198]. Pennyroyal (*Mentha pulegium*) is native to Europe, North Africa, and the Middle East. Another species (*Hedeoma pulegioides*) is native to North America. Pennyroyal is traditionally used in cooking and as an abortifacient. It is also used for colds, pneumonia, and dyspepsia [22,159,199]. Pulegone and menthol cause hepatotoxicity when orally administered to rats [164,167]. Pennyroyal oil causes hepatotoxicity in humans and in mice [159,160,161,162]. Menthone, a component of peppermint oil, caused hepatotoxicity in rats treated with it for 28 days by oral gavage [165]. Pugelone is metabolized to menthofuran by oxidation of the allylic methyl group, followed by an intramolecular cyclization reaction to from a hemiketal, and subsequent dehydration to form a furan [22]. Purified CYP2E1, CYP1A2, and CYP2C19 oxidized pulegone to menthofuran. Then subsequently oxidized menthofuran to 2-hydroxymenthofuran, an intermediate in the formation of mintlactone and isomintlactone [23].

Menthofuran is a monoterpene that is found in several species of mint plants and is oxidized by cytochrome P450 enzymes to hepatotoxic metabolites. The metabolism of menthofuran requires CYP1A2, CYP2E1, and CYP2C19 [22]. These cytochrome P450 enzymes oxidize menthofuran to 2-hydroxymenthofuran, which arises from a dihydrodiol produced from a furan epoxide and is an intermediate in the formation of mintlactone and isomintlactone [22]. The metabolites of menthofuran identified in human and rat liver microsomes that may be responsible for hepatotoxicity, are a γ-ketoenal and epoxides formed by oxidation of the furan ring, which form conjugates with hepatic proteins. CYP1A2, CYP2B6, and CYP3A4 are responsible for the formation of GSH conjugates in human liver fractions [24]. The proteins adducted in rat livers are serum albumin, aldehyde dehydrogenase (ALDH2), malate dehydrogenase (MDH1), and ATP synthase subunit d [166]. The activities of ALDH and ATP complex V were both decreased [166]. Inhibition of cytochrome P450 enzymes with SKF-525A, metyrapone, piperonyl butoxide, and carbon disulfide prevented or reduced the hepatoxicity of pugelone [200].

CYP2A6 is the major cytochrome P450, which is involved in the oxidative metabolism of menthol in human liver microsomes to form (+)-(1*S*,3*S*,4*S*)- and (−)-(1*R*,3*R*,4*R*)-trans-p-methane-3,8-diol derivatives [201]. (−)-Menthol inhibits CYP2A13 and CYP2A6 [32,33]. Additionally, the monoterpene (*R*)-(+)-pulegone causes increases in the marker of liver injury, glutamate pyruvate transaminase, in mice treated with pulegone that are mitigated by pretreatment with disulfiram and cimetidine, inhibitors of CYP2E1 and CYP1A2, respectively [163]. Additionally, menthol may affect the metabolism of pharmaceuticals. Pretreatment of mice with menthol increased the clearance of warfarin and the expression of Cyp2c protein, with a concomitant increase in Car nuclear translocation [202]. Peppermint oil, menthol, and methyl acetate reversibly inhibited the metabolism of nifedipine. Peppermint oil also increased the AUC of felodipine in human liver microsomes containing CYP3A4 [55]. In addition to direct toxicity resulting from pulegone metabolism mediated by the P450 system, components found that peppermint oil, pennyroyal oil, and menthol may directly affect the P450 system, leading to modulations and toxicity of concomitant herbal supplements and pharmaceuticals.

#### 3.3.5. Camphor

Camphor was traditionally prepared from the wood of the camphor laurel (*Cinnamomum camphora*), which is native to Asia. Camphor is now predominantly produced from turpentine and is used in such products as Vicks VapoRub™. Camphor products are usually applied topically to reduce dermatologic complaints and the symptoms of respiratory tract disease [203,204]. (−)-Camphor is a bicyclic monoterpene that is found in the essential oil produced from dalmation sage and is metabolized by CYP2A6 of bacteria overexpressing the human protein [205]. Camphor may be hydroxylated by cytochrome P450 enzymes [206]. In fact, hepatotoxicity of camphor applied topically has been reported in an infant [158]; however, the toxicity may not be ascribable directly to topical administration and could have resulted from unobserved ingestion.

#### 3.3.6. Germander

Germander (*Teucrium chamaedrys* and other species of the genus *Teucrium*) is used to treat gallbladder disease, fever, diarrhea, gout, and for weight loss [207,208,209]. Germander is banned in France, and its use is restricted in the US and Canada [210]. Germander is associated with hepatotoxicity in humans [78,124,125]. The furanoneoclerodane diterpene teucrin A is thought to be responsible for the toxicity of germander, the activation of which in rats [211] and isolated rat hepatocytes [212] led to covalent modification of hepatic proteins. Indeed, pretreatment with phenobarbital, a CAR activator, increased the hepatotoxicity of teurcin A [157], which is activated by cytochrome P450 enzymes and is required for hepatocellular damage in mice. This is concerning because the method of preparation of germander decoctions can affect the concentration of teucrin A [213]. Teucrin A and teuchamaedryn A were found to be hepatotoxic to isolated rat hepatocytes, and this toxicity was decreased by co-treatment with a CYP3A inhibitor [156]. The toxicity was associated with the formation of reactive metabolites that covalently bound hepatic proteins [156]. These proteins were further identified and were found to be mostly of mitochondrial and endoplasmic reticulum origin, and were abducted by the 1,4-enedial derivative of teucrin A to lysine and cysteine residues [211]. The hepatotoxicity of Germander, mediated by teucrin A-adduct formation may be mediated by the P450 system. This is concerning due to reports of hepatotoxicity in humans [61,153,154], as well as its potential use in combinations of other herbal supplements and pharmaceutics that may potentiate adduct formation and subsequent toxicity.

#### 3.3.7. Pyrrolizidine Alkaloids

Pyrrolizidine alkaloids (PAs) and their N-oxides are found in approximately 3% of flowering plants. These plants are predominantly members of the Asteraceae, Boraginaceae, Compositae, and Fabaceae families, and can also be *Senecio*, *Heliotropium*, *Crotalaria*, and *Symphytium* species [214,215]. PAs contain pyrrolizidine, two fused penta rings with nitrogen at position 4. Pyrrole derivatives that react with DNA may cause injury to hepatic vasculature and liver parenchyma [216,217]. (±)-6,7-dihydro-7-hydroxy-1-hydroxymethyl-5H-pyrrolizine–derived DNA adducts cause hepatocarcinoma in rats and are formed from riddelline and monocrotaline by human or rat liver microsomes [218]. Senecionine, monocrotaline, adonifoline, and isoline can be conjugated to glucuronic acid by UGTs in human liver microsomes [219]. 9-Glutahionyl-6,7-dihydro-1-hydroxymethyl-5H-pyrrolizine was detected as conjugated to glutathione in human liver microsomes and in the bile of rats treated that were with the pyrrolizidine alkaloids isoline, retrorsine, and monocrotaline [220]. Monocrotaline caused severe liver and moderate renal toxicity in mice; the degree of toxicity was abrogated by pretreatment with ketoconazole, a CYP3A4 inhibitor [168]. Correspondingly, the presence of N-oxide metabolites of monocrotaline was decreased by pretreatment with ketoconazole. Dehydromonocrotaline and dehydrorectronecine are metabolites of monocrotaline. Dehydromonocrotaline and dehydrorectronecine, but not monocrotaline, caused toxicity in human hepatic sinusoidal endothelial cells and HepG2 cells [168,169]. Retrorsine inhibits the activity of CYP3A4, as measured by testosterone hydroxylation [37]. The hepatotoxicity of PAs and their derivatives is mediated by the P450 system and care should be exercised when herbal supplements containing these compounds are used in combination with other herbal supplements and pharmaceuticals that induce the P450 system.

## 4. Discussion

Herbal supplement use can lead to HILI due to interactions with the P450 system. These interactions may not be predicted until a series of reports of HILI associated with the use of a particular herbal supplement leads to an investigation of potential interactions. Unlike prescription drugs regulated by the FDA (and international agencies), the potential of herbal supplements to induce or inhibit P450 enzymes is not investigated before the supplements are made available to the general population. This lack of information before a supplement is marketed is compounded by the difficulties in event-reporting and history-taking concerning herbal supplement use. Difficulty in assigning an etiologic designation to a particular xenobiotic in patients with positive histories of multi-pharmaceutical, multi-supplement, or mixed pharmaceutical and supplement regimens complicates what “post-marketing” reporting is available. Many networks for reporting exist, but are weakened by difficulties of self-reporting of herbal supplement use by patients and by poor history-taking. However, careful history-taking by physicians may prevent an interaction from taking place and identify the likely offending agent.

Previous articles have discussed herbal supplements and HILI, but this review has focused on herbal supplements that may interact with the P450 system to exacerbate the toxicity of other drugs or to cause toxicity themselves [221,222,223,224]. Herbal supplements may contribute to HILI by inhibiting P450 enzyme activity, inducing P450 gene transcription, or being metabolized by P450 enzymes without affecting their levels or activity. The inhibition of P450 enzymes may contribute to HILI by increasing the exposure of a toxic compound that is metabolized by P450 enzymes. Catechins in green tea, terpenes in black cohosh and cranberry, geniposide and genipin in Gardenia, fucomarins in grapefruit juice, and Echinacea extract may inhibit the activity or decrease the expression of cytochrome P450 enyzmes (Table 1). The induction of P450 genes may contribute to HILI by increasing the exposure of a toxic parent compound that is formed by P450 metabolism or by the release of ROS. Hyperforin in St. John’s Wort, multiple compounds that are found in Gingko biloba, piperine in black and white pepper, diallyl sulfide in garlic, and grapeseed extract increase P450 expression (Table 1 and Table 2). The metabolism of herbal supplements by P450 enzymes may contribute to HILI by resulting in the production of a toxic parent compound. Most of the compounds discussed here are terpenes, pyrrolizidine alkaloids, and ginsenosides.

Careful analysis of existing literature combined with extensive history-taking concerning the use of herbal supplements can help to prevent herb-drug interactions and HILI. The recognition of and reporting of HILI events by physicians can lead to greater knowledge of potential interactions and hepatotoxicity, enabling them to better educate patients of the dangers of some combinations.

## Figures and Tables

**Table 1:** Reports of herbal supplements that inhibit P450 activity or expression.

Herbal Supplement	Preparation/Compound	Effect on P450	CYP450 Reported
Mangosteen (*Garcinia mangostana*)	Aqueous extract	Inhibition of activity [28].	2C8, 2C9, 2C19
Black cohosh (*Actaea racemosa* L. [syn. *Cimifuga racemosa* L.])	Fukinolic acid and cimicfugic acids A and B	Inhibition of activity of purified enzymes [29].	1A2, 2D6, 2C9, 3A4
Green tea * (*Camellia sinensis*)	(−)-epigallocatechin-3-gallate	Inhibition of activity in HLM (human liver micrsomes) and HIM (human intestinal microsomes) [30].	2B6, 2C8, 2C19, 2D6, 3A
	Extract	Inhibition of activity in rat liver microsomes [31].	3a
Menthol	Menthofuran	Inhibited activity in HLM [32].	2A6
	Menthofuran	Inhibited coumarin 7-hyroxylation in purified enzymes [33].	2A6, 2A13
	(−)-menthol *	Inhibited coumarin 7-hydroxylation in purified enzymes [33].	2A13, 2A6
*Garcinia jasminoides* *	Geniposide, extract	Decreased activity in rat liver microsomes [34].	3A4
	Genipin	Inhibited activity and decreased mRNA and protein expression in HepG2 [35].	2C19, 3A4
	Geniposide	Decreased activity in rat livers [34].	3a
Garlic (*Allium sativum*) *	Garlic oil	Inhibited activity reflected by 6-hydroxychlorzoxazone/chlorzaoxazone serum ratios in humans [11,12].	2E1
	Not noted	Inhibited activity reflected by decreased phenacetin metabolism in HLM [36].	1A2
	Retrorsine	Inhibited activity in purified enzymes [37].	3A4
Grapefruit (*Citrus paradisi*) *	Dhydrobergamottin, gergamottin	Inhibited activity in HLM [38,39].	3A4
	Juice	Inhibited activity reflected by decreased midazolam concentrations in humans [39].	3A4
Saw palmetto (*Serenoa repens*)	Extract	Inhibited activity in HLM [40].	2C8
*Echinacea purpura* *	Root Extract (pill)	Inhibition of activity reflected by decreased midazolam hydroxylation in humans [41,42].	3A4
	Root Extract (pill)	Inhibition of activity as reflected by decreased caffeine metabolism in humans [41,42].	1A2
	Marslinic acid, corosolic acid, ursolic acid	Inhibited the activity in HIM [43].	3A4
Cranberry (*vaccinium macrocarpon*)	Extract	Inhibited activity of purified enzymes [44].	1A2, 2D6
Milk thistle (*Silybum marianum*)	Silybin, isosilybin	Decreased mRNA and inhibited PXR-mediated CYP-Luciferase activity in LS180 cell line [45].	3A4
		Inhibited promoter activity via hPXR [45].	3A4
Tomato (*Lycopersicon esculentum*)	Juice extract	Inhibited activity of purified enzymes [46].	3A4
Capsicum (*Capsicum annuum* L. var. grossum.)	Dried and re-suspended in DMSO	Inhibited activity of purified enzymes [46].	3A4
Potato (*Solanum tuberosum* L.)	Dried and re-suspended in DMSO	Inhibited activity of purified enzymes [46].	1A2, 2D6, 3A4
Eggplant (*Solanum melongena* L.)	Dried and re-suspended in DMSO	Inhibited activity of purified enzymes [46].	1A2, 2D6, 3A4
Sweet pepper (*Capsicum annuum*)	Dried and re-suspended in DMSO	Inhibited activity of purified enzymes [46].	1A2, 2D6, 3A4
Black elderberry (*Sambucus nigra*)	Extract (pill)	Inhibited activity in microsomes overexpressing CYP450 [44].	1A2, 2D6, 3A4
Fennel (*Foeniculum vulgare*)	Extract (tea)	Inhibited activity in microsomes overexpressing CYP450 [44].	1A2, 2D6, 3A4
Horsetail (*Equisetaceae family*)	Extract (tea)	Inhibited activity in microsomes overexpressing CYP450 [44].	1A2, 2D6, 3A4
Raspberry leaf (*Rubus idaeus*)	Extract (pill)	Inhibited activity in microsomes overexpressing CYP450 [44].	1A2, 2D6, 3A4
Cinnamon (*Cinnamomum verum*)	*o*-methoxy cinnamaldehyde	Inhibited activity in rat liver microsomes [47].	1a2, 2e1
	Extract	Inhibited activity in microsomes overexpressing CYP450 [48].	2C9, 3A4
Ginger (*Zingiber officinale*)	Extract	Inhibited activity in microsomes overexpressing CYP450 [48].	2C9, 3A4
Mace (*Myristica fragrans*)	Extract	Inhibited activity in microsomes overexpressing CYP450 [48].	2C9, 3A4
Nutmeg (*Myristica genus*)	Extract	Inhibited activity in microsomes overexpressing CYP450 [48].	2C9, 3A4
Valerian (*Valeriana officinalis*)	Extract	Inhibited activity in HLM [40].	2C8
Madagascan medicinal plant (*Catharanthus roseus*)	Ajmalicine	Inhibited activity in HLM [49].	2D6
	Vindolene	Inhibited activity in HLM [49].	2D6, 3A4
	Serpentine	Inhibited activity in HLM [49].	2D6, 3A4
Southern African medicinal plant (*Sutherlandia frutescens*)	Extract	Inhibited activity in transfected microsomes [50].	3A4
Southern African medicinal plant (*Moringa oleifera*)	Extract	Inhibited activity reflected by decreased testosterone hydroxylation in HLM [51].	3A4
West African medicinal plants	Extract	Inhibited activity in transfected microsomes [52].	3A4, 3A5, 3A7
*Aframomum cuspidatum*			
*Aframomum meliguieta*			
*Harrisonia abyssinica*			
*Phyllanthus amarus*			
*Piper guineense*			
*Lonchocarpus sericeus*			
*Lipia multiflora*			
West African medicinal plants	Extract	Inhibited activity in transfected microsomes [52].	3A4, 3A7
*Jutropha curcas*			
*Persia Americana*			
*Oxytenanthera abyssinica*			
*Talinum triangulare*			
Tanzanian medicinal plant			
*Cyphostemma ildebrandtii*			
Tanzanian medicinal plant (*Acacia nilotica*)	Extract	Inhibited activity in transfected microsomes [53,54].	2C9, 2C19, 2D6, 3A4
Tanzanian medicinal plants	Extract	Inhibited activity in transfected microsomes [53,54].	2C9, 2C19, 3A4
*Acacia robusta*			
*Agauria salicifolia*			
Tanzanian medicinal plants	Extract	Inhibited activity in transfected microsomes [53,54].	2C9, 3A4
*Elaeodendron buchananii*			
*Sclerocarya birrea*			
Peppermint (*Mentha piperita*)	Oil	Inhibited activity in HLM [55].	3A4
	Menthol *	Inhibited activity in HLM [55].	3A4
	Menthyl acetate	Inhibited activity in HLM [55].	3A4
	Ascorbyl palmitate	Inhibited activity in HLM [55].	3A4
Sesamin (*Sesamum indicum*)	Not noted	Inhibited activity reflected by decreased phenacetin, diclofenac, omeprazole, dextromethorphan, and midazolam metabolism in HLM [36].	1A2, 2C9, 2C19, 2D6, 3A4
Tumeric (*Curcuma longa*)	Not noted	Inhibited activity reflected by decreased diclofenac, omeprazole, dextromethorphan, and midazolam metabolism in HLM [36].	2C9, 2C19, 2D6, 3A4
St. John’s wort (Hypericum perforatum)	Not noted	Inhibited activity reflected by decreased phenacetin, diclofenac, and midazolam metabolism in HLM [36].	1A2, 2C9, 3A4

Herbs, compounds, or preparations marked with an asterisk have been associated with alteration of P450 metabolism in human subjects or have been associated with hepatotoxicity and may be clinically significant.

**Table 2:** Reports of herbal supplements with human Pregnane X Receptor (hPXR)-dependent or -independent induction of P450 expression.

Herbal Supplement	Preparation/Compound	Effect on hPXR
Gan Gao-Licorice (Radix et Rhizoma Glycyrrhizae)	Extract	Activation of CYP3A4 promoter via hPXR [96].
Huang Qi-*Astragalus mebranaceus* (Radix Astragali)	Extract	Activation of CYP3A4 promoter via hPXR [96].
Ji Xue Cao-*Centella asiatica* (Herba Centellae)	Extract	Activation of CYP3A4 promoter via hPXR [96].
Ban Lan Gen-*Isatis indigotica* (Radix Isatidis)	Extract	Activation of CYP3A4 promoter via hPXR [96].
Jin Yin Hua-*Lonicera japonica* (Flos Lonicerae Japonicae)	Extract	Activation of CYP3A4 promoter via hPXR [96].
Hong Jing Tian-*Rhodiola crenulata* (Radix et Rhizoma Rhodiolae Crenulatae)	Extract	Activation of CYP3A4 promoter via hPXR [96].
Da Huang-Rhubarb (Radix et Rhizoma Rhei)	Extract	Activation of CYP3A4 promoter via hPXR [96].
	Trans-resveratrol	Activation of CYP3A4 promoter via hPXR [96].
Fu Ling-*Poria cocos* (Poria)	Extract	Activation of CYP3A4 promoter via hPXR [96].
Bai Shao-*Paeonia lactiflora* (Radix Paeoniae Alba)	Extract	Activation of CYP3A4 promoter via hPXR [96].
Sang Qi-*Panax notoginseng* (Radix et Rhizoma Notoginseng)	Extract *	Activation of CYP3A4 promoter via hPXR [96].
Chuan Xiong-*Ligusticum chuanxiong* (Rhizoma Chuanxiong)	Extract	Activation of CYP3A4 promoter via hPXR [96].
Dang Gui-Chinese angelica (Radix *Angelicae sinensis*)	Extract	Activation of CYP3A4 promoter via hPXR [96].
	Ligustilide	Activation of CYP3A4 promoter via hPXR [96].
Sheng Di Huang-Rehmannia root (Radix Rehmanniae)	Extract	Activation of CYP3A4 promoter via hPXR [96].
Yin Yang Huo-*Epimedium brevicornum* (Herba Epimedii)	Extract	Activation of CYP3A4 promoter via hPXR [96].
Di Gu Pi-*Lycium chinense* (Cortex Lycii)	Extract	Activation of CYP3A4 promoter via hPXR [96].
Bai Zhu-*Atractylodes macrocephala* (Rhizoma Atractylodis)	Extract	Activation of CYP3A4 promoter via hPXR [96].
Wu Wei Zi-*Schisandra chinensis* (*Fructus* Schisandrae Chinensis)	Extract	Activation of CYP3A4 promoter via hPXR [96].
	Schisantherin A	Activation of CYP3A4 promoter via hPXR [96].
Bai Shao-*Paeonia lactiflora* (Radix Paeoniae Alba)	Extract	Activation of CYP3A4 promoter via hPXR [96].
Mai Dong-*Ophiopogon japonicas* (Radix Ophiopogonis)	Extract	Activation of CYP3A4 promoter via hPXR [96].
Hu Zhang-*Polygonum multiflorum* (Radix Polygoni Multiflori)	Extract	Activation of CYP3A4 promoter via hPXR [96].
Huang Lian-*Coptis chinensis* (Rhizoma Coptidis)	Extract	Activation of CYP3A4 promoter via hPXR [96].
	Berberine hydrochloride	Activation of CYP3A4 promoter via hPXR [96].
Yin Chen-*Artemisia scoparia* (Herba Artemisiae Scopariae)	Extract	Activation of CYP3A4 promoter via hPXR [96].
Tian Hua Fen-*Trichosanthes kirilowii* (Radix Trichosanthis)	Extract	Activation of CYP3A4 promoter via hPXR [96].
Shui Fei Ji-*Silybum marianum* (*Fructus silybi*)	Extract	Activation of CYP3A4 promoter via hPXR [96].
Zhi Zi-Gardenia fruit (*Fructus gardeniae*)	Extract *	Activation of CYP3A4 promoter via hPXR [96].
Ren Shen–Ginseng (Radix et Rhizoma *ginseng*) *	Ginsenoside F2, protopanaxadiol	Activation of CYP3A4 promoter via hPXR [97].
	Panaxatriol, Rg2, pseudoginsenoside F11, Rg1, ginsenodide, Rb3	Activation of CYP3A4 promoter via hPXR [97].
	Extract	Activation of CYP3A4 promoter via hPXR [96].
Black pepper (*Piper nigrum*) *	Piperine	Activation of CYP3A4 promoter via hPXR, increased mRNA and protein in intestinal cell lines and human hepatocytes [98].
St. John’s Wort * (*Hypericum perforatum*)	Hyperforin	Activation of CYP3A4 promoter via hPXR [99,100].
	Hyperforin	Increased CYP2C9 and 3A4 protein and mRNA in human hepatocytes [99,100].
	Extract (pill)	Increased CYP3A4 activity reflected by decreased phenytoin concentrations [12].
		Increased CYP2C19 activity reflected by decreased omeprazole concentrations in humans dependent on CYP2C19 phenotype [13,14].
		Increased activity reflected by hydroxymidazolam/midazolam serum ratios in humans (CYP3A4) 6-hydroxychlorzoxazone/chlorzaoxazone serum ratios in humans (CYP2E1) [11,12].
*Gingko biloba* *	Extract	Activation of CYP3A4 promoter via hPXR [96,101].
	Gingkolide A, Gingkolide B	Activation of CYP3A4 promoter via hPXR [102].
		Increased CYP2B6 and 3A4 mRNA in PHH [102].
	Leaf extract	Increased activity of CYP2C19 reflected by decreased plasma concentrations of omeprazole and increased 5-hydroxyomeprazole in humans [103].
Kava Kava * (*Piper methysticum*)	Extract	Activation of CYP3A4 promoter via hPXR [104].
	Desmethoxyangonin, dihydromethysticin	Increased Cyp3a23 mRNA in rat livers [105].
*Echinacea purpura* *	Extract	Activation of CYP3A4 promoter via hPXR [87].
	Extract	Increased CYP1A2 and 3A4 mRNA in HepG2 [87].
	Extract	Increased CYP1A2 and 3A4 mRNA in HepG2 [87].
Thyme (*Thymus vulgaris*)	Extract	Activation of CYP3A4 promoter via hPXR [106].
Clove (*Syzygium aromaticum*)	Extract	Activation of CYP3A4 promoter via hPXR [106].
Tumeric (*Curcuma longa*)	Curcumin	Activation of CYP3A4 promoter via hPXR [106].
Red wine (*Vitis vinifera*)	Resveratrol	Activation of CYP3A4 promoter via hPXR [106].
Southern African medicinal plant (*Hypoxis hemerocallidea*)	Extract	Activation of CYP3A4 promoter via hPXR [50].
	Rooperol	Activation of CYP3A4 promoter via hPXR [50].
	Stimasterol	Activation of CYP3A4 promoter via hPXR [50].
Southern African medicinal plant (*Sutherlandia frutescens*)	Extract	Activation of CYP3A4 promoter via hPXR [50].
Tanzanian medicinal plant (*Cyphostemma hildebrandtii*)	Extract	Activation of CYP3A4 promoter via hPXR [53,54].
Tanzanian medicinal plant (*Agauria salicifolia*)	Extract	Activation of CYP3A4 promoter via hPXR [53,54].
Tanzanian medicinal plant (*Elaeodendron buchananii*)	Extract	Activation of CYP3A4 promoter via hPXR [53,54].
Tanzanian medicinal plant (*Sclerocarya birrea*)	Extract	Activation of CYP3A4 promoter via hPXR [53,54].
Tanzanian medicinal plant (*Sterculia Africana*)	Extract	Activation of CYP3A4 promoter via hPXR [53,54].
Tanzanian medicinal plant (*Turraea holstii*)	Extract	Activation of CYP3A4 promoter via hPXR [53,54].
Allspice (*Pimenta dioica*)	Extract	Increased transcription of CYP3A4 in HepG2 cell line [106].
Grape seed (*Vitis vinifera*)	Extract	Increased CYP3A4 mRNA in PHH [104].
Garlic (*Allium sativum*) *	Diallysulfide	Increased Cyp2b1 and 2b2 mRNA in rat livers [107].

Herbs, compounds or preparations marked with an asterisk have been associated with alteration of P450 metabolism in human subjects or have been associated with hepatotoxicity and may be clinically significant.

**Table 3:** Reported Hepatotoxicity of Herbal Supplements.

Herbal Supplement	Preparation/Compound	Toxicity
Black cohosh (*Actaea racemosa*)	Extract	Liver necrosis, autoimmune hepatitis, protein adducts [69,70].
Ginseng (*Panax ginseng* and *P*. *quinquefolius*)	Extract	Possible liver injury in a patient after interaction with imatinib [134].
Greater celandine (*Chelidonium* majus)	Extract	Reports of hepatocellular injury in humans [148,149,150,151].
		Single report of cholestasis in human [148].
Black Pepper (*Piper nigrum*)	Piperine	Increased liver enzymes with CCl_4_ and hepatic lipid peroxidation in mice [10].
St. John’s Wort (*Hypericum perforatum*)	Extract	Liver injury associated with copaiba use [125].
	Extract	Increased toxicity associated with tert-butyl hydroperoxide [152].
Green tea (*Camellia sinensis*)	Epigallocatechin-3-gallate	Hepatotoxic in mice [64].
	Extract	Hepatotoxic in rats [65].
Germander (Lamiaceae family, *Teucrim* Genus)		Hepatotoxic in humans [61,153,154,155].
	Teucrin A, teuchamaedryn A	Hepatotoxic to isolated rat hepatocytes, CYP3A4 dependent [156].
	Teucrin A	Hepatocellular toxicity in mice [157].
*Gingko biloba*	Extract	May be hepatotoxic in mice [130].
	Extract	Hepatotoxic in humans [61].
Camphor (*Cinnamomum camphora*)	Topical cream	Single report of hepatotoxicity in a human [158].
Kava kava (*Piper methysticum*)	Extract	Hepatotoxic in humans [61].
Pennyroyal oil (*Mentha pulegium* and *Hedeoma pulegioides*)	Oil	Hepatotoxic in mice [159].
	Oil	Hepatotoxic in humans [160,161,162].
	(*R*)-(+)-pulegone	CYP2E1/1A2-dependent hepatotoxicity in mice [163].
	Pulegone, menthol	Hepatotoxic in mice [164].
	Menthone	Hepatotoxic in rats [165,166].
	Pulegone, menthol	Hepatotoxic in rats [164,167].
*Gardenia* (*Fructus gardenia*)	Extract (30% geniposide)	Hepatotoxic in rats [91].
	Geniposide	Hepatotoxic in rats [92].
	Extract	Hepatotoxic in rats [92].
Garlic (*Allium sativum*)	Homogenate	Hepatotoxic in rats [144,145].
Found in multiple species of plants	Monocrotaline	CYP3A4-dependent hepatotoxicity in mice [168].
	Dehydromonocrotaline, dehydrorectronecine	Toxic to human hepatoma cell lines [168,169].
*Garcinia cambogia*	Extract	Hepatotoxic to humans [170].
Mistletoe (*Viscum coloratum*)	Extract	Hepatotoxic to humans [171].

## Abbreviations

mRNA: messenger ribonucleic acid; DNA: deoxyribonucleic acid; CYP450: cytochrome P450; MDR1: multiple drug resistance 1; UTI: urinary tract infection; US: United States; FDA: Food and Drug Administration; PXR: pregnane X receptor; CAR/Car: constitutive androstane receptor; AST: aspartate transaminase; ALT: alanine transaminase; HIV: human immunodeficiency virus; DILI: drug-induced liver injury; HILI: herb-induced liver injury UGT: Uridine glucuronosyltransferase; MRP2: multiple resistance protein 2; AUC: area under the curve; WT: wild-type; ATP: adenosine triphosphate; ROS: reactive oxygen species; GGT: γ-glutamyl transferase; CIOMS: The Council for International Organizations of Medical Sciences; DILIN: drug induced liver injury network; PA: pyrrolizidine alkaloids; WHO: World Health Organization; GI: gastrointestinal; PHH: primary human hepatocytes; HIM: human intestinal microsomes.

## References

[1] Tucker G.T., Houston J.B., Huang S.M. (2001). Optimizing drug development: Strategies to assess drug metabolism/transporter interaction potential-toward a consensus. Clin. Pharmacol. Ther..

[2] Matthews H.B., Lucier G.W., Fisher K.D. (1999). Medicinal herbs in the United States: Research needs. Environ. Health Perspect..

[3] Bessone F., Hernandez N., Lucena M.I., Andrade R.J., Latin Dili Network L., Spanish Dili R. (2016). The latin american DILI registry experience: A successful ongoing collaborative strategic initiative. Int. J. Mol. Sci..

[4] Hayashi P.H. (2016). Drug-induced liver injury network causality assessment: Criteria and experience in the United States. Int. J. Mol. Sci..

[5] Misaka S., Miyazaki N., Fukushima T., Yamada S., Kimura J. (2013). Effects of green tea extract and (−)-epigallocatechin-3-gallate on pharmacokinetics of nadolol in rats. Phytomedicine.

[6] Kliewer S.A., Moore J.T., Wade L., Staudinger J.L., Watson M.A., Jones S.A., McKee D.D., Oliver B.B., Willson T.M., Zetterstrom R.H. (1998). An orphan nuclear receptor activated by pregnanes defines a novel steroid signaling pathway. Cell.

[7] Lehmann J.M., McKee D.D., Watson M.A., Willson T.M., Moore J.T., Kliewer S.A. (1998). The human orphan nuclear receptor PXR is activated by compounds that regulate *CYP3A4* gene expression and cause drug interactions. J. Clin. Investig..

[8] Cherian M.T., Chai S.C., Chen T. (2015). Small-molecule modulators of the constitutive androstane receptor. Expert Opin. Drug Metab. Toxicol..

[9] Honkakoski P., Zelko I., Sueyoshi T., Negishi M. (1998). The nuclear orphan receptor CAR-retinoid X receptor heterodimer activates the phenobarbital-responsive enhancer module of the *CYP2B* gene. Mol. Cell. Biol..

[10] Piyachaturawat P., Kingkaeohoi S., Toskulkao C. (1995). Potentiation of carbon tetrachloride hepatotoxicity by piperine. Drug Chem. Toxicol..

[11] Gurley B.J., Gardner S.F., Hubbard M.A., Williams D.K., Gentry W.B., Cui Y., Ang C.Y. (2005). Clinical assessment of effects of botanical supplementation on cytochrome P450 phenotypes in the elderly: St John’s wort, garlic oil, panax ginseng and ginkgo biloba. Drugs Aging.

[12] Gurley B.J., Gardner S.F., Hubbard M.A., Williams D.K., Gentry W.B., Cui Y., Ang C.Y. (2002). Cytochrome P450 phenotypic ratios for predicting herb-drug interactions in humans. Clin. Pharmacol. Ther..

[13] Wang L.S., Zhou G., Zhu B., Wu J., Wang J.G., Abd El-Aty A.M., Li T., Liu J., Yang T.L., Wang D. (2004). St John’s wort induces both cytochrome P450 3A4-catalyzed sulfoxidation and 2C19-dependent hydroxylation of omeprazole. Clin. Pharmacol. Ther..

[14] Wang L.S., Zhu B., Abd El-Aty A.M., Zhou G., Li Z., Wu J., Chen G.L., Liu J., Tang Z.R., An W. (2004). The influence of St John’s wort on CYP2C19 activity with respect to genotype. J. Clin. Pharmacol..

[15] Raji M.A., Kuo Y.F., Snih S.A., Sharaf B.M., Loera J.A. (2005). Ethnic differences in herb and vitamin/mineral use in the elderly. Ann. Pharmacother..

[16] Bruno J.J., Ellis J.J. (2005). Herbal use among us elderly: 2002 national health interview survey. Ann. Pharmacother..

[17] Dergal J.M., Gold J.L., Laxer D.A., Lee M.S., Binns M.A., Lanctot K.L., Freedman M., Rochon P.A. (2002). Potential interactions between herbal medicines and conventional drug therapies used by older adults attending a memory clinic. Drugs Aging.

[18] Canter P.H., Ernst E. (2004). Herbal supplement use by persons aged over 50 years in britain: Frequently used herbs, concomitant use of herbs, nutritional supplements and prescription drugs, rate of informing doctors and potential for negative interactions. Drugs Aging.

[19] Verma S., Thuluvath P.J. (2007). Complementary and alternative medicine in hepatology: Review of the evidence of efficacy. Clin. Gastroenterol. Hepatol..

[20] Sanfelix Genoves J., Palop Larrea V., Rubio Gomis E., Martinez-Mir I. (2001). Consumption of medicinal herbs and medicines. Aten. Primaria.

[21] Stjernberg L., Berglund J., Halling A. (2006). Age and gender effect on the use of herbal medicine products and food supplements among the elderly. Scand. J. Prim. Health Care.

[22] Gordon W.P., Huitric A.C., Seth C.L., McClanahan R.H., Nelson S.D. (1987). The metabolism of the abortifacient terpene, (*R*)-(+)-pulegone, to a proximate toxin, menthofuran. Drug Metab. Dispos..

[23] Khojasteh-Bakht S.C., Chen W., Koenigs L.L., Peter R.M., Nelson S.D. (1999). Metabolism of (*R*)-(+)-pulegone and (*R*)-(+)-menthofuran by human liver cytochrome P-450s: Evidence for formation of a furan epoxide. Drug Metab. Dispos..

[24] Lassila T., Mattila S., Turpeinen M., Pelkonen O., Tolonen A. (2016). Tandem mass spectrometric analysis of S- and N-linked glutathione conjugates of pulegone and menthofuran and identification of P450 enzymes mediating their formation. Rapid Commun. Mass Spectrom..

[25] Tsintis P., La Mache E. (2004). Cioms and ich initiatives in pharmacovigilance and risk management: Overview and implications. Drug Saf..

[26] Ioannides C., Lewis D.F. (2004). Cytochromes P450 in the bioactivation of chemicals. Curr. Top. Med. Chem..

[27] Brewer C.T., Chen T. (2016). PXR variants: The impact on drug metabolism and therapeutic responses. Acta Pharm. Sin. B.

[28] Foti R.S., Pearson J.T., Rock D.A., Wahlstrom J.L., Wienkers L.C. (2009). In vitro inhibition of multiple cytochrome P450 isoforms by xanthone derivatives from mangosteen extract. Drug Metab. Dispos..

[29] Huang Y., Jiang B., Nuntanakorn P., Kennelly E.J., Shord S., Lawal T.O., Mahady G.B. (2010). Fukinolic acid derivatives and triterpene glycosides from black cohosh inhibit CYP isozymes, but are not cytotoxic to HEP-G2 cells in vitro. Curr. Drug Saf..

[30] Misaka S., Kawabe K., Onoue S., Werba J.P., Giroli M., Tamaki S., Kan T., Kimura J., Watanabe H., Yamada S. (2013). Effects of green tea catechins on cytochrome P450 2B6, 2C8, 2C19, 2D6 and 3A activities in human liver and intestinal microsomes. Drug Metab. Pharmacokinet..

[31] Misaka S., Kawabe K., Onoue S., Werba J.P., Giroli M., Watanabe H., Yamada S. (2013). Green tea extract affects the cytochrome P450 3A activity and pharmacokinetics of simvastatin in rats. Drug Metab. Pharmacokinet..

[32] Yamaguchi Y., Akimoto I., Motegi K., Yoshimura T., Wada K., Nishizono N., Oda K. (2013). Synthetic models related to methoxalen and menthofuran-cytochrome P450 (CYP) 2A6 interactions. Benzofuran and coumarin derivatives as potent and selective inhibitors of CYP2A6. Chem. Pharm. Bull..

[33] Kramlinger V.M., von Weymarn L.B., Murphy S.E. (2012). Inhibition and inactivation of cytochrome P450 2A6 and cytochrome P450 2A13 by menthofuran, beta-nicotyrine and menthol. Chem. Biol. Interact..

[34] Kang J.J., Wang H.W., Liu T.Y., Chen Y.C., Ueng T.H. (1997). Modulation of cytochrome P-450-dependent monooxygenases, glutathione and glutathione s-transferase in rat liver by geniposide from gardenia jasminoides. Food Chem. Toxicol..

[35] Gao L.N., Zhang Y., Cui Y.L., Yan K. (2014). Evaluation of genipin on human cytochrome P450 isoenzymes and P-glycoprotein in vitro. Fitoterapia.

[36] Sasaki T., Sato Y., Kumagai T., Yoshinari K., Nagata K. (2017). Effect of health foods on cytochrome P450-mediated drug metabolism. J. Pharm. Health Care Sci..

[37] Dai J., Zhang F., Zheng J. (2010). Retrorsine, but not monocrotaline, is a mechanism-based inactivator of P450 3A4. Chem. Biol. Interact..

[38] Messer A., Raquet N., Lohr C., Schrenk D. (2012). Major furocoumarins in grapefruit juice ii: Phototoxicity, photogenotoxicity, and inhibitory potency vs. Cytochrome P450 3A4 activity. Food Chem. Toxicol..

[39] Tanaka S., Uchida S., Miyakawa S., Inui N., Takeuchi K., Watanabe H., Namiki N. (2013). Comparison of inhibitory duration of grapefruit juice on organic anion-transporting polypeptide and cytochrome P450 3A4. Biol. Pharm. Bull..

[40] Albassam A.A., Mohamed M.E., Frye R.F. (2015). Inhibitory effect of six herbal extracts on CYP2C8 enzyme activity in human liver microsomes. Xenobiotica.

[41] Gorski J.C., Huang S.M., Pinto A., Hamman M.A., Hilligoss J.K., Zaheer N.A., Desai M., Miller M., Hall S.D. (2004). The effect of echinacea (echinacea purpurea root) on cytochrome P450 activity in vivo. Clin. Pharmacol. Ther..

[42] Gurley B.J., Gardner S.F., Hubbard M.A., Williams D.K., Gentry W.B., Carrier J., Khan I.A., Edwards D.J., Shah A. (2004). In vivo assessment of botanical supplementation on human cytochrome P450 phenotypes: Citrus aurantium, echinacea purpurea, milk thistle, and saw palmetto. Clin. Pharmacol. Ther..

[43] Kim E., Sy-Cordero A., Graf T.N., Brantley S.J., Paine M.F., Oberlies N.H. (2011). Isolation and identification of intestinal CYP3A inhibitors from cranberry (*Vaccinium macrocarpon*) using human intestinal microsomes. Planta Med..

[44] Langhammer A.J., Nilsen O.G. (2014). In vitro inhibition of human CYP1A2, CYP2D6, and CYP3A4 by six herbs commonly used in pregnancy. Phytother. Res..

[45] Mooiman K.D., Maas-Bakker R.F., Moret E.E., Beijnen J.H., Schellens J.H., Meijerman I. (2013). Milk thistle’s active components silybin and isosilybin: Novel inhibitors of PXR-mediated CYP3A4 induction. Drug Metab. Dispos..

[46] Sunaga K., Ohkawa K., Nakamura K., Ohkubo A., Harada S., Tsuda T. (2012). Mechanism-based inhibition of recombinant human cytochrome P450 3A4 by tomato juice extract. Biol. Pharm. Bull..

[47] Hasegawa A., Yoshino M., Nakamura H., Ishii I., Watanabe T., Kiuchi M., Ishikawa T., Ohmori S., Kitada M. (2002). Identification of inhibitory component in cinnamon—*O*-methoxycinnamaldehyde inhibits CYP1A2 and CYP2E1. Drug Metab. Pharmacokinet..

[48] Kimura Y., Ito H., Hatano T. (2010). Effects of mace and nutmeg on human cytochrome P450 3A4 and 2C9 activity. Biol. Pharm. Bull..

[49] Usia T., Watabe T., Kadota S., Tezuka Y. (2005). Cytochrome P450 2D6 (CYP2D6) inhibitory constituents of catharanthus roseus. Biol. Pharm. Bull..

[50] Mills E., Foster B.C., van Heeswijk R., Phillips E., Wilson K., Leonard B., Kosuge K., Kanfer I. (2005). Impact of african herbal medicines on antiretroviral metabolism. AIDS.

[51] Monera T.G., Wolfe A.R., Maponga C.C., Benet L.Z., Guglielmo J. (2008). *Moringa oleifera* leaf extracts inhibit 6beta-hydroxylation of testosterone by CYP3A4. J. Infect. Dev. Ctries..

[52] Agbonon A., Eklu-Gadegbeku K., Aklikokou K., Gbeassor M., Akpagana K., Tam T.W., Arnason J.T., Foster B.C. (2010). In vitro inhibitory effect of west african medicinal and food plants on human cytochrome P450 3A subfamily. J. Ethnopharmacol..

[53] Deferme S., Kamuhabwa A., Nshimo C., de Witte P., Augustijns P. (2003). Screening of tanzanian plant extracts for their potential inhibitory effect on P-glycoprotein mediated efflux. Phytother. Res..

[54] Van den Bout-van den Beukel C.J., Hamza O.J., Moshi M.J., Matee M.I., Mikx F., Burger D.M., Koopmans P.P., Verweij P.E., Schoonen W.G., van der Ven A.J. (2008). Evaluation of cytotoxic, genotoxic and CYP450 enzymatic competition effects of tanzanian plant extracts traditionally used for treatment of fungal infections. Basic Clin. Pharmacol. Toxicol..

[55] Dresser G.K., Wacher V., Wong S., Wong H.T., Bailey D.G. (2002). Evaluation of peppermint oil and ascorbyl palmitate as inhibitors of cytochrome P450 3A4 activity in vitro and in vivo. Clin. Pharmacol. Ther..

[56] Sueoka N., Suganuma M., Sueoka E., Okabe S., Matsuyama S., Imai K., Nakachi K., Fujiki H. (2001). A new function of green tea: Prevention of lifestyle-related diseases. Ann. N. Y. Acad. Sci..

[57] Dona M., Dell’Aica I., Calabrese F., Benelli R., Morini M., Albini A., Garbisa S. (2003). Neutrophil restraint by green tea: Inhibition of inflammation, associated angiogenesis, and pulmonary fibrosis. J. Immunol..

[58] Raederstorff D.G., Schlachter M.F., Elste V., Weber P. (2003). Effect of EGCG on lipid absorption and plasma lipid levels in rats. J. Nutr. Biochem..

[59] Sartippour M.R., Shao Z.M., Heber D., Beatty P., Zhang L., Liu C., Ellis L., Liu W., Go V.L., Brooks M.N. (2002). Green tea inhibits vascular endothelial growth factor (VEGF) induction in human breast cancer cells. J. Nutr..

[60] Haqqi T.M., Anthony D.D., Gupta S., Ahmad N., Lee M.S., Kumar G.K., Mukhtar H. (1999). Prevention of collagen-induced arthritis in mice by a polyphenolic fraction from green tea. Proc. Natl. Acad. Sci. USA.

[61] Teschke R., Wolff A., Frenzel C., Schulze J., Eickhoff A. (2012). Herbal hepatotoxicity: A tabular compilation of reported cases. Liver Int..

[62] Mazzanti G., Di Sotto A., Vitalone A. (2015). Hepatotoxicity of green tea: An update. Arch. Toxicol..

[63] Yao H.T., Hsu Y.R., Lii C.K., Lin A.H., Chang K.H., Yang H.T. (2014). Effect of commercially available green and black tea beverages on drug-metabolizing enzymes and oxidative stress in wistar rats. Food Chem. Toxicol..

[64] Wang D., Wang Y., Wan X., Yang C.S., Zhang J. (2015). Green tea polyphenol (−)-epigallocatechin-3-gallate triggered hepatotoxicity in mice: Responses of major antioxidant enzymes and the NRF2 rescue pathway. Toxicol. Appl. Pharmacol..

[65] Emoto Y., Yoshizawa K., Kinoshita Y., Yuki M., Yuri T., Yoshikawa Y., Sayama K., Tsubura A. (2014). Green tea extract-induced acute hepatotoxicity in rats. J. Toxicol. Pathol..

[66] Teschke R., Zhang L., Melzer L., Schulze J., Eickhoff A. (2014). Green tea extract and the risk of drug-induced liver injury. Expert Opin. Drug Metab. Toxicol..

[67] Gentry-Maharaj A., Karpinskyj C., Glazer C., Burnell M., Ryan A., Fraser L., Lanceley A., Jacobs I., Hunter M.S., Menon U. (2015). Use and perceived efficacy of complementary and alternative medicines after discontinuation of hormone therapy: A nested United Kingdom collaborative trial of ovarian cancer screening cohort study. Menopause.

[68] Su Y., Wu L., Wang Q., Yang B., Kuang H. (2014). New 9,19-cycloartenol glycosides isolated from the roots of cimicifuga simplex and their anti-inflammatory effects. Bioorg. Med. Chem. Lett..

[69] Lim T.Y., Considine A., Quaglia A., Shawcross D.L. (2013). Subacute liver failure secondary to black cohosh leading to liver transplantation. BMJ Case Rep..

[70] Enbom E.T., Le M.D., Oesterich L., Rutgers J., French S.W. (2014). Mechanism of hepatotoxicity due to black cohosh (*Cimicifuga racemosa*): Histological, immunohistochemical and electron microscopy analysis of two liver biopsies with clinical correlation. Exp. Mol. Pathol..

[71] Franco D.L., Kale S., Lam-Himlin D.M., Harrison M.E. (2017). Black cohosh hepatotoxicity with autoimmune hepatitis presentation. Case Rep. Gastroenterol..

[72] Teschke R. (2010). Black cohosh and suspected hepatotoxicity: Inconsistencies, confounding variables, and prospective use of a diagnostic causality algorithm. A critical review. Menopause.

[73] Yokotani K., Chiba T., Sato Y., Nakanishi T., Murata M., Umegaki K. (2013). Effect of three herbal extracts on cytochrome P450 and possibility of interaction with drugs. Shokuhin Eiseigaku Zasshi.

[74] Walton E.W. (2014). Topical phytochemicals: Applications for wound healing. Adv. Skin Wound Care.

[75] Schell J., Betts N.M., Foster M., Scofield R.H., Basu A. (2017). Cranberries improve postprandial glucose excursions in type 2 diabetes. Food Funct..

[76] Avorn J., Monane M., Gurwitz J.H., Glynn R.J., Choodnovskiy I., Lipsitz L.A. (1994). Reduction of bacteriuria and pyuria after ingestion of cranberry juice. JAMA.

[77] Moore G.A. (2001). Oranges and lemons: Clues to the taxonomy of citrus from molecular markers. Trends Genet..

[78] Cingi C., Toros S.Z., Gurbuz M.K., Ince I., Cakli H., Erdogmus N., Karasulu E., Kaya E. (2013). Effect of grapefruit juice on bioavailability of montelukast. Laryngoscope.

[79] Chanet A., Milenkovic D., Deval C., Potier M., Constans J., Mazur A., Bennetau-Pelissero C., Morand C., Berard A.M. (2012). Naringin, the major grapefruit flavonoid, specifically affects atherosclerosis development in diet-induced hypercholesterolemia in mice. J. Nutr. Biochem..

[80] Cirmi S., Maugeri A., Ferlazzo N., Gangemi S., Calapai G., Schumacher U., Navarra M. (2017). Anticancer potential of citrus juices and their extracts: A systematic review of both preclinical and clinical studies. Front. Pharmacol..

[81] Lee A.J., Chan W.K., Harralson A.F., Buffum J., Bui B.C. (1999). The effects of grapefruit juice on sertraline metabolism: An in vitro and in vivo study. Clin. Ther..

[82] De la Garza A.L., Etxeberria U., Haslberger A., Aumueller E., Martinez J.A., Milagro F.I. (2015). Helichrysum and grapefruit extracts boost weight loss in overweight rats reducing inflammation. J. Med. Food.

[83] Holmberg M.T., Tornio A., Neuvonen M., Neuvonen P.J., Backman J.T., Niemi M. (2014). Grapefruit juice inhibits the metabolic activation of clopidogrel. Clin. Pharmacol. Ther..

[84] Yotsawimonwat S., Rattanadechsakul J., Rattanadechsakul P., Okonogi S. (2010). Skin improvement and stability of echinacea purpurea dermatological formulations. Int. J. Cosmet. Sci..

[85] Mousa H.A. (2017). Prevention and treatment of influenza, influenza-like illness, and common cold by herbal, complementary, and natural therapies. J. Evid.-Based Complement. Altern. Med..

[86] Smejkal K., Rjaskova V. (2016). Use of plant extracts as an efficient alternative therapy of respiratory tract infections. Ceska a Slovenska Farmacie.

[87] Awortwe C., Manda V.K., Avonto C., Khan S.I., Khan I.A., Walker L.A., Bouic P.J., Rosenkranz B. (2015). *Echinacea purpurea* up-regulates CYP1A2, CYP3A4 and MDR1 gene expression by activation of pregnane X receptor pathway. Xenobiotica.

[88] Wu R., Tao W., Zhang H., Xue W., Zou Z., Wu H., Cai B., Doron R., Chen G. (2016). Instant and persistent antidepressant response of gardenia yellow pigment is associated with acute protein synthesis and delayed upregulation of BDNF expression in the hippocampus. ACS Chem. Neurosci..

[89] Appiah S., Revitt M., Jones H., Vu M., Simmonds M., Bell C. (2017). Antiinflammatory and hepatoprotective medicinal herbs as potential substitutes for bear bile. Int. Rev. Neurobiol..

[90] Higashino S., Sasaki Y., Giddings J.C., Hyodo K., Sakata S.F., Matsuda K., Horikawa Y., Yamamoto J. (2014). Crocetin, a carotenoid from gardenia jasminoides ellis, protects against hypertension and cerebral thrombogenesis in stroke-prone spontaneously hypertensive rats. Phytother. Res..

[91] Yamano T., Tsujimoto Y., Noda T., Shimizu M., Ohmori M., Morita S., Yamada A. (1988). Hepatotoxicity of gardenia yellow color in rats. Toxicol. Lett..

[92] Yang H.J., Fu M.H., Wu Z.L., Liang R.X., Huang L.Q., Fang J., Li G., Cao Y. (2006). Experimental studies on hepatotoxicity of rats induced by *Fructus gardeniae*. Zhongguo Zhong Yao Za Zhi.

[93] Wei J., Zhang F., Zhang Y., Cao C., Li X., Li D., Liu X., Yang H., Huang L. (2014). Proteomic investigation of signatures for geniposide-induced hepatotoxicity. J. Proteome Res..

[94] Kumar S., Jin M., Ande A., Sinha N., Silverstein P.S., Kumar A. (2012). Alcohol consumption effect on antiretroviral therapy and HIV-1 pathogenesis: Role of cytochrome P450 isozymes. Expert Opin. Drug Metab. Toxicol..

[95] Pal D., Kwatra D., Minocha M., Paturi D.K., Budda B., Mitra A.K. (2011). Efflux transporters- and cytochrome P-450-mediated interactions between drugs of abuse and antiretrovirals. Life Sci..

[96] Yu C., Chai X., Yu L., Chen S., Zeng S. (2011). Identification of novel pregnane X receptor activators from traditional chinese medicines. J. Ethnopharmacol..

[97] Wang Y.G., Liu H.S., Zhang X.X., Xiao Y., Lu B.B., Ma Z.C., Liang Q.D., Tang X.L., Xiao C.R., Tan H.L. (2013). Screening of pregnane X receptor activation from ginsenosides. Yao Xue Xue Bao.

[98] Wang Y.M., Lin W., Chai S.C., Wu J., Ong S.S., Schuetz E.G., Chen T. (2013). Piperine activates human pregnane x receptor to induce the expression of cytochrome P450 3A4 and multidrug resistance protein 1. Toxicol. Appl. Pharmacol..

[99] Chen Y., Ferguson S.S., Negishi M., Goldstein J.A. (2004). Induction of human CYP2C9 by rifampicin, hyperforin, and phenobarbital is mediated by the pregnane x receptor. J. Pharmacol. Exp. Ther..

[100] Godtel-Armbrust U., Metzger A., Kroll U., Kelber O., Wojnowski L. (2007). Variability in PXR-mediated induction of CYP3A4 by commercial preparations and dry extracts of St. John’s wort. Naunyn Schmiedebergs Arch. Pharmacol..

[101] Yeung E.Y., Sueyoshi T., Negishi M., Chang T.K. (2008). Identification of ginkgo biloba as a novel activator of pregnane x receptor. Drug Metab. Dispos..

[102] Li L., Stanton J.D., Tolson A.H., Luo Y., Wang H. (2009). Bioactive terpenoids and flavonoids from ginkgo biloba extract induce the expression of hepatic drug-metabolizing enzymes through pregnane X receptor, constitutive androstane receptor, and aryl hydrocarbon receptor-mediated pathways. Pharm. Res..

[103] Yin O.Q., Tomlinson B., Waye M.M., Chow A.H., Chow M.S. (2004). Pharmacogenetics and herb-drug interactions: Experience with ginkgo biloba and omeprazole. Pharmacogenetics.

[104] Raucy J.L. (2003). Regulation of CYP3A4 expression in human hepatocytes by pharmaceuticals and natural products. Drug Metab. Dispos..

[105] Ma Y., Sachdeva K., Liu J., Ford M., Yang D., Khan I.A., Chichester C.O., Yan B. (2004). Desmethoxyyangonin and dihydromethysticin are two major pharmacological kavalactones with marked activity on the induction of CYP3A23. Drug Metab. Dispos..

[106] Kluth D., Banning A., Paur I., Blomhoff R., Brigelius-Flohe R. (2007). Modulation of pregnane X receptor- and electrophile responsive element-mediated gene expression by dietary polyphenolic compounds. Free Radic. Biol. Med..

[107] Zhang P., Noordine M.L., Cherbuy C., Vaugelade P., Pascussi J.M., Duee P.H., Thomas M. (2006). Different activation patterns of rat xenobiotic metabolism genes by two constituents of garlic. Carcinogenesis.

[108] Watkins R.E., Maglich J.M., Moore L.B., Wisely G.B., Noble S.M., Davis-Searles P.R., Lambert M.H., Kliewer S.A., Redinbo M.R. (2003). 2.1 a crystal structure of human PXR in complex with the St. John’s wort compound hyperforin. Biochemistry.

[109] Zollner G., Wagner M., Trauner M. (2010). Nuclear receptors as drug targets in cholestasis and drug-induced hepatotoxicity. Pharmacol. Ther..

[110] Chen T. (2008). Nuclear receptor drug discovery. Curr. Opin. Chem. Biol..

[111] Wang Y.M., Ong S.S., Chai S.C., Chen T. (2012). Role of CAR and PXR in xenobiotic sensing and metabolism. Expert Opin. Drug Metab. Toxicol..

[112] Aleksunes L.M., Klaassen C.D. (2012). Coordinated regulation of hepatic phase i and ii drug-metabolizing genes and transporters using AhR-, CAR-, PXR-, PPARα-, and Nrf2-null mice. Drug Metab. Dispos..

[113] Gardner-Stephen D., Heydel J.M., Goyal A., Lu Y., Xie W., Lindblom T., Mackenzie P., Radominska-Pandya A. (2004). Human PXR variants and their differential effects on the regulation of human UDP-glucuronosyltransferase gene expression. Drug Metab. Dispos..

[114] Oladimeji P.O., Lin W., Brewer C.T., Chen T. (2017). Glucose-dependent regulation of pregnane x receptor is modulated by AMP-activated protein kinase. Sci. Rep..

[115] Lamba V., Yasuda K., Lamba J.K., Assem M., Davila J., Strom S., Schuetz E.G. (2004). PXR (NR1I2): Splice variants in human tissues, including brain, and identification of neurosteroids and nicotine as PXR activators. Toxicol. Appl. Pharmacol..

[116] Gong H., Singh S.V., Singh S.P., Mu Y., Lee J.H., Saini S.P., Toma D., Ren S., Kagan V.E., Day B.W. (2006). Orphan nuclear receptor pregnane X receptor sensitizes oxidative stress responses in transgenic mice and cancerous cells. Mol. Endocrinol..

[117] Ma X., Shah Y., Cheung C., Guo G.L., Feigenbaum L., Krausz K.W., Idle J.R., Gonzalez F.J. (2007). The pregnane X receptor gene-humanized mouse: A model for investigating drug-drug interactions mediated by cytochromes P450 3A. Drug Metab. Dispos..

[118] Baes M., Gulick T., Choi H.S., Martinoli M.G., Simha D., Moore D.D. (1994). A new orphan member of the nuclear hormone receptor superfamily that interacts with a subset of retinoic acid response elements. Mol. Cell. Biol..

[119] Sugatani J., Kojima H., Ueda A., Kakizaki S., Yoshinari K., Gong Q.H., Owens I.S., Negishi M., Sueyoshi T. (2001). The phenobarbital response enhancer module in the human bilirubin UDP-glucuronosyltransferase UGT1A1 gene and regulation by the nuclear receptor CAR. Hepatology.

[120] Huang W., Zhang J., Chua S.S., Qatanani M., Han Y., Granata R., Moore D.D. (2003). Induction of bilirubin clearance by the constitutive androstane receptor (CAR). Proc. Natl. Acad. Sci. USA.

[121] Kawamoto T., Sueyoshi T., Zelko I., Moore R., Washburn K., Negishi M. (1999). Phenobarbital-responsive nuclear translocation of the receptor CAR in induction of the CYP2b gene. Mol. Cell. Biol..

[122] Moore L.B., Parks D.J., Jones S.A., Bledsoe R.K., Consler T.G., Stimmel J.B., Goodwin B., Liddle C., Blanchard S.G., Willson T.M. (2000). Orphan nuclear receptors constitutive androstane receptor and pregnane X receptor share xenobiotic and steroid ligands. J. Biol. Chem..

[123] Maglich J.M., Parks D.J., Moore L.B., Collins J.L., Goodwin B., Billin A.N., Stoltz C.A., Kliewer S.A., Lambert M.H., Willson T.M. (2003). Identification of a novel human constitutive androstane receptor (CAR) agonist and its use in the identification of CAR target genes. J. Biol. Chem..

[124] Stojanovic N.M., Samardzic L., Randjelovic P.J., Radulovic N.S. (2017). Prevalence of self-medication practice with herbal products among non-psychotic psychiatric patients from southeastern serbia: A cross-sectional study. Saudi Pharm. J..

[125] Agollo M.C., Miszputen S.J., Diament J. (2014). Hypericum perforatum-induced hepatotoxicity with possible association with copaiba (*Copaifera langsdorffii Desf*): Case report. Einstein.

[126] Evans J.R. (2013). Ginkgo biloba extract for age-related macular degeneration. Cochrane Database Syst. Rev..

[127] Abdel-Zaher A.O., Farghaly H.S.M., El-Refaiy A.E.M., Abd-Eldayem A.M. (2017). Protective effect of the standardized extract of ginkgo biloba (EGB761) against hypertension with hypercholesterolemia-induced renal injury in rats: Insights in the underlying mechanisms. Biomed. Pharmacother..

[128] Coelho C., Tyler R., Ji H., Rojas-Roncancio E., Witt S., Tao P., Jun H.J., Wang T.C., Hansen M.R., Gantz B.J. (2016). Survey on the effectiveness of dietary supplements to treat tinnitus. Am. J. Audiol..

[129] Zhang H.F., Huang L.B., Zhong Y.B., Zhou Q.H., Wang H.L., Zheng G.Q., Lin Y. (2016). An overview of systematic reviews of ginkgo biloba extracts for mild cognitive impairment and dementia. Front. Aging Neurosci..

[130] Maeda J., Inoue K., Ichimura R., Takahashi M., Kodama Y., Saito N., Yoshida M. (2015). Essential role of constitutive androstane receptor in ginkgo biloba extract induced liver hypertrophy and hepatocarcinogenesis. Food Chem. Toxicol..

[131] Reay J.L., Scholey A.B., Kennedy D.O. (2010). Panax ginseng (G115) improves aspects of working memory performance and subjective ratings of calmness in healthy young adults. Hum. Psychopharmacol..

[132] Scholey A., Ossoukhova A., Owen L., Ibarra A., Pipingas A., He K., Roller M., Stough C. (2010). Effects of american ginseng (*Panax quinquefolius*) on neurocognitive function: An acute, randomised, double-blind, placebo-controlled, crossover study. Psychopharmacology.

[133] Gum S.I., Jo S.J., Ahn S.H., Kim S.G., Kim J.T., Shin H.M., Cho M.K. (2007). The potent protective effect of wild ginseng (*Panax ginseng* C.A. Meyer) against benzo[α]pyrene-induced toxicity through metabolic regulation of CYP1A1 and GSTs. J. Ethnopharmacol..

[134] Bilgi N., Bell K., Ananthakrishnan A.N., Atallah E. (2010). Imatinib and panax ginseng: A potential interaction resulting in liver toxicity. Ann. Pharmacother..

[135] Bajad S., Bedi K.L., Singla A.K., Johri R.K. (2001). Antidiarrhoeal activity of piperine in mice. Planta Med..

[136] Chiu N.T., Tomlinson Guns E.S., Adomat H., Jia W., Deb S. (2014). Identification of human cytochrome P450 enzymes involved in the hepatic and intestinal biotransformation of 20(*S*)-protopanaxadiol. Biopharm. Drug Dispos..

[137] Prasad S., Tyagi A.K. (2016). Historical spice as a future drug: Therapeutic potential of piperlongumine. Curr. Pharm. Des..

[138] Ziment I. (1991). History of the treatment of chronic bronchitis. Respiration.

[139] De Souza Grinevicius V.M., Kviecinski M.R., Santos Mota N.S., Ourique F., Porfirio Will Castro L.S., Andreguetti R.R., Gomes Correia J.F., Filho D.W., Pich C.T., Pedrosa R.C. (2016). Piper nigrum ethanolic extract rich in piperamides causes ROS overproduction, oxidative damage in DNA leading to cell cycle arrest and apoptosis in cancer cells. J. Ethnopharmacol..

[140] Lee G.H., Bhandary B., Lee E.M., Park J.K., Jeong K.S., Kim I.K., Kim H.R., Chae H.J. (2011). The roles of ER stress and P450 2E1 in CCL(4)-induced steatosis. Int. J. Biochem. Cell Biol..

[141] Stjernberg L., Berglund J. (2000). Garlic as an insect repellent. JAMA.

[142] Fisher C.D., Augustine L.M., Maher J.M., Nelson D.M., Slitt A.L., Klaassen C.D., Lehman-McKeeman L.D., Cherrington N.J. (2007). Induction of drug-metabolizing enzymes by garlic and allyl sulfide compounds via activation of constitutive androstane receptor and nuclear factor E2-related factor 2. Drug Metab. Dispos..

[143] Sueyoshi T., Green W.D., Vinal K., Woodrum T.S., Moore R., Negishi M. (2011). Garlic extract diallyl sulfide (DAS) activates nuclear receptor CAR to induce the sult1e1 gene in mouse liver. PLoS ONE.

[144] Rana S.V., Pal R., Vaiphei K., Singh K. (2006). Garlic hepatotoxicity: Safe dose of garlic. Trop. Gastroenterol..

[145] Oboh G. (2006). Tropical green leafy vegetables prevent garlic-induced hepatotoxicity in the rat. J. Med. Food.

[146] Letsyo E., Jerz G., Winterhalter P., Lindigkeit R., Beuerle T. (2017). Incidence of pyrrolizidine alkaloids in herbal medicines from german retail markets: Risk assessments and implications to consumers. Phytother. Res..

[147] Lin Y.P., Zhang M.P., Wang K.Y., Sun C.Y., Wang Y. (2016). Research achievements on ginsenosides biosynthesis from panax ginseng. Zhongguo Zhong Yao Za Zhi.

[148] Stickel F., Poschl G., Seitz H.K., Waldherr R., Hahn E.G., Schuppan D. (2003). Acute hepatitis induced by greater celandine (*Chelidonium majus*). Scand. J. Gastroenterol..

[149] Hardeman E., Van Overbeke L., Ilegems S., Ferrante M. (2008). Acute hepatitis induced by greater celandine (*Chelidonium majus*). Acta Gastroenterol. Belg..

[150] Teschke R., Glass X., Schulze J. (2011). Herbal hepatotoxicity by greater celandine (*Chelidonium majus*): Causality assessment of 22 spontaneous reports. Regul. Toxicol. Pharmacol..

[151] Teschke R., Frenzel C., Glass X., Schulze J., Eickhoff A. (2012). Greater celandine hepatotoxicity: A clinical review. Ann. Hepatol..

[152] Valentao P., Carvalho M., Carvalho F., Fernandes E., das Neves R.P., Pereira M.L., Andrade P.B., Seabra R.M., Bastos M.L. (2004). Hypericum androsaemum infusion increases *tert*-butyl hydroperoxide-induced mice hepatotoxicity in vivo. J. Ethnopharmacol..

[153] Dag M., Ozturk Z., Aydnl M., Koruk I., Kadayfc A. (2014). Postpartum hepatotoxicity due to herbal medicine teucrium polium. Ann. Saudi Med..

[154] Sezer R.G., Bozaykut A. (2012). Pediatric hepatotoxicity associated with polygermander (*Teucrium polium*). Clin. Toxicol..

[155] Goksu E., Kilic T., Yilmaz D. (2012). Hepatitis: A herbal remedy germander. Clin. Toxicol..

[156] Lekehal M., Pessayre D., Lereau J.M., Moulis C., Fouraste I., Fau D. (1996). Hepatotoxicity of the herbal medicine germander: Metabolic activation of its furano diterpenoids by cytochrome P450 3A depletes cytoskeleton-associated protein thiols and forms plasma membrane blebs in rat hepatocytes. Hepatology.

[157] Kouzi S.A., McMurtry R.J., Nelson S.D. (1994). Hepatotoxicity of germander (*Teucrium chamaedrys* L.) and one of its constituent neoclerodane diterpenes teucrin a in the mouse. Chem. Res. Toxicol..

[158] Teschke R. (2010). Kava hepatotoxicity—A clinical review. Ann. Hepatol..

[159] Gordon P., Khojasteh S.C. (2015). A decades-long investigation of acute metabolism-based hepatotoxicity by herbal constituents: A case study of pennyroyal oil. Drug Metab. Rev..

[160] Anderson I.B., Mullen W.H., Meeker J.E., Khojasteh Bakht S.C., Oishi S., Nelson S.D., Blanc P.D. (1996). Pennyroyal toxicity: Measurement of toxic metabolite levels in two cases and review of the literature. Ann. Intern. Med..

[161] Gordon W.P., Forte A.J., McMurtry R.J., Gal J., Nelson S.D. (1982). Hepatotoxicity and pulmonary toxicity of pennyroyal oil and its constituent terpenes in the mouse. Toxicol. Appl. Pharmacol..

[162] Bakerink J.A., Gospe S.M., Dimand R.J., Eldridge M.W. (1996). Multiple organ failure after ingestion of pennyroyal oil from herbal tea in two infants. Pediatrics.

[163] Sztajnkrycer M.D., Otten E.J., Bond G.R., Lindsell C.J., Goetz R.J. (2003). Mitigation of pennyroyal oil hepatotoxicity in the mouse. Acad. Emerg. Med..

[164] Thorup I., Wurtzen G., Carstensen J., Olsen P. (1983). Short term toxicity study in rats dosed with pulegone and menthol. Toxicol. Lett..

[165] Madsen C., Wurtzen G., Carstensen J. (1986). Short-term toxicity study in rats dosed with menthone. Toxicol. Lett..

[166] Khojasteh S.C., Hartley D.P., Ford K.A., Uppal H., Oishi S., Nelson S.D. (2012). Characterization of rat liver proteins adducted by reactive metabolites of menthofuran. Chem. Res. Toxicol..

[167] Thorup I., Wurtzen G., Carstensen J., Olsen P. (1983). Short term toxicity study in rats dosed with peppermint oil. Toxicol. Lett..

[168] Yao J., Li C.G., Gong L.K., Feng C.C., Li C.Z., Gao M., Luan Y., Qi X.M., Ren J. (2014). Hepatic cytochrome P450s play a major role in monocrotaline-induced renal toxicity in mice. Acta Pharmacol. Sin..

[169] Yang M., Ruan J., Fu P.P., Lin G. (2016). Cytotoxicity of pyrrolizidine alkaloid in human hepatic parenchymal and sinusoidal endothelial cells: Firm evidence for the reactive metabolites mediated pyrrolizidine alkaloid-induced hepatotoxicity. Chem. Biol. Interact..

[170] Corey R., Werner K.T., Singer A., Moss A., Smith M., Noelting J., Rakela J. (2016). Acute liver failure associated with garcinia cambogia use. Ann. Hepatol..

[171] Kim H.J., Kim H., Ahn J.H., Suk H.J. (2015). Liver injury induced by herbal extracts containing mistletoe and kudzu. J. Altern. Complement. Med..

[172] Wang Y.M., Chai S.C., Brewer C.T., Chen T. (2014). Pregnane X receptor and drug-induced liver injury. Expert Opin. Drug Metab. Toxicol..

[173] Woolbright B.L., Jaeschke H. (2012). Novel insight into mechanisms of cholestatic liver injury. World J. Gastroenterol..

[174] Schyschka L., Sanchez J.J., Wang Z., Burkhardt B., Muller-Vieira U., Zeilinger K., Bachmann A., Nadalin S., Damm G., Nussler A.K. (2013). Hepatic 3D cultures but not 2D cultures preserve specific transporter activity for acetaminophen-induced hepatotoxicity. Arch. Toxicol..

[175] Navarro V.J., Senior J.R. (2006). Drug-related hepatotoxicity. N. Engl. J. Med..

[176] Suk K.T., Kim D.J., Kim C.H., Park S.H., Yoon J.H., Kim Y.S., Baik G.H., Kim J.B., Kweon Y.O., Kim B.I. (2012). A prospective nationwide study of drug-induced liver injury in korea. Am. J. Gastroenterol..

[177] Chalasani N., Fontana R.J., Bonkovsky H.L., Watkins P.B., Davern T., Serrano J., Yang H., Rochon J. (2008). Drug Induced Liver Injury Network. Causes, clinical features, and outcomes from a prospective study of drug-induced liver injury in the united states. Gastroenterology.

[178] Meier Y., Pauli-Magnus C., Zanger U.M., Klein K., Schaeffeler E., Nussler A.K., Nussler N., Eichelbaum M., Meier P.J., Stieger B. (2006). Interindividual variability of canalicular ATP-binding-cassette (ABC)-transporter expression in human liver. Hepatology.

[179] Suzuki N., Irie M., Iwata K., Nakane H., Yoshikane M., Koyama Y., Uehara Y., Takeyama Y., Kitamura Y., Sohda T. (2006). Altered expression of alkaline phosphatase (ALP) in the liver of primary biliary cirrhosis (PBC) patients. Hepatol. Res..

[180] Fisher K., Vuppalanchi R., Saxena R. (2015). Drug-induced liver injury. Arch. Pathol. Lab. Med..

[181] Teschke R., Larrey D., Melchart D., Danan G. (2016). Traditional chinese medicine (TCM) and herbal hepatotoxicity: Rucam and the role of novel diagnostic biomarkers such as microRNAs. Medicines.

[182] Kumar B.S., Chung B.C., Kwon O.S., Jung B.H. (2012). Discovery of common urinary biomarkers for hepatotoxicity induced by carbon tetrachloride, acetaminophen and methotrexate by mass spectrometry-based metabolomics. J. Appl. Toxicol..

[183] Xie Y., McGill M.R., Dorko K., Kumer S.C., Schmitt T.M., Forster J., Jaeschke H. (2014). Mechanisms of acetaminophen-induced cell death in primary human hepatocytes. Toxicol. Appl. Pharmacol..

[184] Li F., Lu J., Cheng J., Wang L., Matsubara T., Csanaky I.L., Klaassen C.D., Gonzalez F.J., Ma X. (2013). Human PXR modulates hepatotoxicity associated with rifampicin and isoniazid co-therapy. Nat. Med..

[185] Ganey P.E., Luyendyk J.P., Maddox J.F., Roth R.A. (2004). Adverse hepatic drug reactions: Inflammatory episodes as consequence and contributor. Chem. Biol. Interact..

[186] Ganey P.E., Luyendyk J.P., Newport S.W., Eagle T.M., Maddox J.F., Mackman N., Roth R.A. (2007). Role of the coagulation system in acetaminophen-induced hepatotoxicity in mice. Hepatology.

[187] Tujios S., Fontana R.J. (2011). Mechanisms of drug-induced liver injury: From bedside to bench. Nat. Rev. Gastroenterol. Hepatol..

[188] Den Braver M.W., den Braver-Sewradj S.P., Vermeulen N.P., Commandeur J.N. (2016). Characterization of cytochrome P450 isoforms involved in sequential two-step bioactivation of diclofenac to reactive P-benzoquinone imines. Toxicol. Lett..

[189] Kishida T., Onozato T., Kanazawa T., Tanaka S., Kuroda J. (2012). Increase in covalent binding of 5-hydroxydiclofenac to hepatic tissues in rats co-treated with lipopolysaccharide and diclofenac: Involvement in the onset of diclofenac-induced idiosyncratic hepatotoxicity. J. Toxicol. Sci..

[190] Lundgren H., Martinsson K., Cederbrant K., Jirholt J., Mucs D., Madeyski-Bengtson K., Havarinasab S., Hultman P. (2017). HLA-DR7 and HLA-DQ2: Transgenic mouse strains tested as a model system for ximelagatran hepatotoxicity. PLoS ONE.

[191] Clare K.E., Miller M.H., Dillon J.F. (2017). Genetic factors influencing drug-induced liver injury: Do they have a role in prevention and diagnosis?. Curr. Hepatol. Rep..

[192] Ali N., Auerbach H.E. (2017). New-onset acute thrombocytopenia in hospitalized patients: Pathophysiology and diagnostic approach. J. Community Hosp. Intern. Med. Perspect..

[193] Ghannam M., Mansour S., Nabulsi A., Abdoh Q. (2017). Anticonvulsant hypersensitivity syndrome after phenytoin administration in an adolescent patient: A case report and review of literature. Clin. Mol. Allergy.

[194] Benichou C. (1990). Standardization of definitions and criteria of causality assessment of adverse drug reactions. Drug-induced liver disorders: Report of an international consensus meeting. Int. J. Clin. Pharmacol. Ther. Toxicol..

[195] Danan G., Teschke R. (2015). Rucam in drug and herb induced liver injury: The update. Int. J. Mol. Sci..

[196] Benichou C., Danan G., Flahault A. (1993). Causality assessment of adverse reactions to drugs—II. An original model for validation of drug causality assessment methods: Case reports with positive rechallenge. J. Clin. Epidemiol..

[197] Danan G., Benichou C. (1993). Causality assessment of adverse reactions to drugs—I. A novel method based on the conclusions of international consensus meetings: Application to drug-induced liver injuries. J. Clin. Epidemiol..

[198] Yamazaki F., Sone R. (2017). Desensitization of menthol-activated cold receptors in lower extremities during local cooling in young women with a cold constitution. J. Physiol. Sci..

[199] Ciganda C., Laborde A. (2003). Herbal infusions used for induced abortion. J. Toxicol. Clin. Toxicol..

[200] Mizutani T., Nomura H., Nakanishi K., Fujita S. (1987). Effects of drug metabolism modifiers on pulegone-induced hepatotoxicity in mice. Res. Commun. Chem. Pathol. Pharmacol..

[201] Miyazawa M., Marumoto S., Takahashi T., Nakahashi H., Haigou R., Nakanishi K. (2011). Metabolism of (+)- and (−)-menthols by CYP2A6 in human liver microsomes. J. Oleo Sci..

[202] Hoshino M., Ikarashi N., Tsukui M., Kurokawa A., Naito R., Suzuki M., Yokobori K., Ochiai T., Ishii M., Kusunoki Y. (2014). Menthol reduces the anticoagulant effect of warfarin by inducing cytochrome P450 2C expression. Eur. J. Pharm. Sci..

[203] Paul I.M. (2012). Therapeutic options for acute cough due to upper respiratory infections in children. Lung.

[204] Hoy N.Y., Leung A.K., Metelitsa A.I., Adams S. (2012). New concepts in median nail dystrophy, onychomycosis, and hand, foot, and mouth disease nail pathology. ISRN Dermatol..

[205] Nakahashi H., Miyazawa M. (2011). Biotransformation of (−)-camphor by salmonella typhimurium OY1002/2A6 expressing human CYP2A6 and NADPH-P450 reductase. J. Oleo Sci..

[206] Jerome S.V., Hughes T.F., Friesner R.A. (2016). Successful application of the DBLOC method to the hydroxylation of camphor by cytochrome P450. Protein Sci..

[207] Herrera S., Bruguera M. (2008). Hepatotoxicity induced by herbs and medicines used to induce weight loss. Gastroenterol. Hepatol..

[208] Jaradat N.A., Ayesh O.I., Anderson C. (2016). Ethnopharmacological survey about medicinal plants utilized by herbalists and traditional practitioner healers for treatments of diarrhea in the west bank/palestine. J. Ethnopharmacol..

[209] Guesmi F., Prasad S., Tyagi A.K., Landoulsi A. (2017). Antinflammatory and anticancer effects of terpenes from oily fractions of teucruim alopecurus, blocker of IκBα kinase, through downregulation of nf-kappab activation, potentiation of apoptosis and suppression of NF-κB-regulated gene expression. Biomed. Pharmacother..

[210] Bosisio E., Giavarini F., Dell’Agli M., Galli G., Galli C.L. (2004). Analysis by high-performance liquid chromatography of teucrin A in beverages flavoured with an extract of *Teucrium chamaedrys* L.. Food Addit. Contam..

[211] Druckova A., Mernaugh R.L., Ham A.J., Marnett L.J. (2007). Identification of the protein targets of the reactive metabolite of teucrin a in vivo in the rat. Chem. Res. Toxicol..

[212] Fau D., Lekehal M., Farrell G., Moreau A., Moulis C., Feldmann G., Haouzi D., Pessayre D. (1997). Diterpenoids from germander, an herbal medicine, induce apoptosis in isolated rat hepatocytes. Gastroenterology.

[213] Nencini C., Galluzzi P., Pippi F., Menchiari A., Micheli L. (2014). Hepatotoxicity of *Teucrium chamaedrys* L. Decoction: Role of difference in the harvesting area and preparation method. Indian J. Pharmacol..

[214] Smith L.W., Culvenor C.C. (1981). Plant sources of hepatotoxic pyrrolizidine alkaloids. J. Nat. Prod..

[215] Ridker P.M., Ohkuma S., McDermott W.V., Trey C., Huxtable R.J. (1985). Hepatic venocclusive disease associated with the consumption of pyrrolizidine-containing dietary supplements. Gastroenterology.

[216] Valla D., Benhamou J.P. (1988). Drug-induced vascular and sinusoidal lesions of the liver. Baillieres Clin. Gastroenterol..

[217] Bye S.N., Dutton M.F. (1991). The inappropriate use of traditional medicines in south africa. J. Ethnopharmacol..

[218] He X., Xia Q., Ma L., Fu P.P. (2016). 7-cysteine-pyrrole conjugate: A new potential DNA reactive metabolite of pyrrolizidine alkaloids. J. Environ. Sci. Health C Environ. Carcinog. Ecotoxicol. Rev..

[219] He Y.Q., Yang L., Liu H.X., Zhang J.W., Liu Y., Fong A., Xiong A.Z., Lu Y.L., Yang L., Wang C.H. (2010). Glucuronidation, a new metabolic pathway for pyrrolizidine alkaloids. Chem. Res. Toxicol..

[220] Chen M., Li L., Zhong D., Shen S., Zheng J., Chen X. (2016). 9-glutathionyl-6,7-dihydro-1-hydroxymethyl-5 h-pyrrolizine is the major pyrrolic glutathione conjugate of retronecine-type pyrrolizidine alkaloids in liver microsomes and in rats. Chem. Res. Toxicol..

[221] Sarges P., Steinberg J.M., Lewis J.H. (2016). Drug-induced liver injury: Highlights from a review of the 2015 literature. Drug Saf..

[222] Lee W.J., Kim H.W., Lee H.Y., Son C.G. (2015). Systematic review on herb-induced liver injury in korea. Food Chem. Toxicol..

[223] Chen M., Suzuki A., Borlak J., Andrade R.J., Lucena M.I. (2015). Drug-induced liver injury: Interactions between drug properties and host factors. J. Hepatol..

[224] Shahbaz O., Mahajan S., Lewis J.H. (2017). Highlights of drug—And herb-induced liver injury in the literature from 2016: How best to translate new information into clinical practice?. Expert Opin. Drug Metab. Toxicol..

